# A multi-scale remote sensing semantic segmentation model with boundary enhancement based on UNetFormer

**DOI:** 10.1038/s41598-025-99663-9

**Published:** 2025-04-27

**Authors:** JiangQing Wang, Ting Chen, Lu Zheng, Jun Tie, YiBo Zhang, PinTing Chen, ZhiQing Luo, QuanJie Song

**Affiliations:** 1 College of Computer Science, South-Central Minzu University, Wuhan, 430074 Hubei China; 2 Hubei Provincial Engineering Research Center for Intelligent Management of Manufacturing Enterprises, Wuhan, 430074 China; 3 Hubei Provincial Engineering Research Center of Agricultural Blockchain and Intelligent Management, Wuhan, 430074 China; 4 Hubei Academy of Scientific and Technical Information, Wuhan, 430071 China; 5 Institute of Agricultural Economics and Technology, Hubei Academy of Agricultural Sciences, Wuhan, 430064 Hubei China

**Keywords:** Computational science, Computer science, Environmental sciences

## Abstract

The precise execution of semantic segmentation on remote sensing data is a pivotal factor. It determines the achievements and impact of geoscience endeavors and their applications. However, challenges caused by target edge blurring and scale variability in high-resolution remote sensing imagery hinder the improvement of segmentation accuracy. In this work, to address these issues, a Boundary-Enhanced Multi-Scale Semantic Segmentation Network (BEMS-UNetFormer) based on UNetFormer is proposed for remote sensing data. Firstly, an improved Boundary Awareness Module (BAM) is used to extract the edge information of the target from the low-level features to enhance the recognition of the target edges. Secondly, the improved Boundary-Guided Fusion Module (BFM) incorporates the edge information from BAM into subsequent decoding, further refining the precise representation of boundary regions. Finally, at the pivotal junction between the encoder and decoder, the Multi-Scale Cascaded Atrous Spatial Pyramid Pooling (MSC-ASPP) is designed, capable of deeply mining and integrating multi-scale deep features. The method was tested on two mainstream datasets, Potsdam and Vaihingen, achieving 86.12% and 83.10% MIoU, respectively, improving by 1.38% and 1.79% over the baseline model. Notably, the IoU and F1 Score for the small-scale target “Car” in the Potsdam dataset reached 91.20% and 95.57%, respectively, while the “Building” and “LowVeg” categories in the Vaihingen dataset achieved the highest IoU and F1 Score. The experimental results indicate that the proposed method demonstrates higher precision in segmenting small-scale targets and target boundaries, surpassing mainstream methods overall.

## Introduction

Remote sensing semantic segmentation aims to assign each pixel in a remote sensing image to a semantic category, forming a segmentation map to achieve the recognition and extraction of different objects and regions on the ground. It is widely used in many fields, such as urban planning, land use, precision agriculture, environmental monitoring, and disaster assessment.

With the rapid advancements in deep learning, remote sensing semantic segmentation methods based on deep learning have emerged as the dominant approach. These methods can be categorized into three groups: methods based on convolutional neural networks (CNN)^1^, Transformer-based methods^2^, and Mamba-based methods^3^.

The first category is based on Convolutional Neural Networks. Fully Convolutional Networks (FCNs) proposed by Long et al.^4^ recovers resolution and detail through backward convolution and skipping. Badrinarayanan et al.^5^ proposed SegNet, which introduces an encoder-decoder structure into image semantic segmentation, achieving end-to-end pixel-level segmentation. The DeepLab family of networks^6–8^ utilizes Atrous Spatial Pyramid Pooling (ASPP) with varying receptive fields to achieve multi-scale feature extraction. However, this method results in the loss of fine details, leading to coarser segmentation outcomes. Zhao et al.^9^ proposed the PSPNet, which focuses on capturing homogeneous contextual dependencies but ignores category differences, leading to unreliable context when confusing categories are present in the scenes. Ronneberger et al.^10^ proposed UNet, which uses a symmetrical U-shaped structure and skip connections to fuse the features of the encoder with the corresponding decoder features. In recent years, the Attention Mechanism has been widely applied in the field of image semantic segmentation, enabling the adaptive selection of key information from complex features. Li et al.^11^ proposed the Gated Channel Transformation (GCT), which, through gating mechanisms and normalization, effectively suppresses redundant information and avoids noise interference during feature learning. Hou et al.^12^ introduced Coordinate Attention, which captures spatial position correlations in feature maps alongside orientation-aware and position-sensitive information, thereby enabling models to locate and identify target objects more accurately.

The second category focuses on Transformer-based methods. Although Convolutional Neural Networks achieve strong performance in image semantic segmentation, CNN-based models struggle to capture global contextual information over long distances^13^. This results in reduced segmentation accuracy for small-sized objects and those with high interclass similarity. Transformers construct long-range dependencies by stacking multiple attention modules, thereby strengthening feature representation. Zheng et al.^14^ proposed SETR, inspired by the Vision Transformer (ViT)^15^, leveraging the ViT encoder’s Transformer layers for global context modeling and designing three decoders for image semantic segmentation, but at the cost of increased parameters and computational complexity. Liu et al.^16^ proposed the Swin Transformer, which adopts a sliding window design based on ViT, significantly reducing computational complexity while maintaining performance. Recent research has focused on integrating CNNs and Transformers to fully extract local and global information from images. He et al.^17^ proposed the ST-UNet for remote sensing image semantic segmentation, embedding the Swin Transformer into the UNet to achieve efficient fusion of global and local information, but it exhibits limitations in accurately segmenting edge regions. Wang et al.^18^ proposed UNetFormer, an efficient semantic segmentation model for remote sensing urban scene images, combining the characteristics of UNet and Transformer to effectively capture and represent semantic information in complex urban scenes.

The third category encompasses Mamba-based methods. Recently, Albert et al.^3^ proposed Mamba, a novel selective state space model that demonstrates strong performance in long sequence modeling tasks. Mamba integrates the recurrent nature of RNNs, the parallel computation and attention mechanisms of Transformers, and the linear properties of State Space Models (SSMs)^19^. Compared with traditional CNNs and ViTs, Mamba can efficiently capture global semantic information while significantly reducing computational complexity. Zhu et al.^20^ proposed Samba, which introduces the Mamba architecture into the semantic segmentation of remote sensing images by designing an encoder structure, Samba Block, tailored for high-resolution remote sensing images. Samba Block combines Mamba Block and Multilayer Perceptron (MLP) and is designed to efficiently extract features from image sequences while maintaining computational efficiency. However, Samba with ViT, which lacks sufficient focus on local details, results in false-negative segmentation errors. Ma et al.^21^ proposed the RS3Mamba network architecture, which enhances the performance of the convolution-based main branch by constructing a dual-branch network with the VSS module to provide global information. Zhu et al.^22^ proposed UNetMamba, which includes a CNN-based encoder for extracting local image features and a Mamba-based decoder for aggregating and integrating global information, thus enabling accurate and efficient semantic segmentation of remote sensing images.

Based on the above analysis, although the deep learning-based semantic segmentation methods for remote sensing images have achieved remarkable results, there are still some urgent problems to address. These issues arise from the current research status of remote sensing semantic segmentation and the characteristics of high-resolution remote sensing data. Most mainstream semantic segmentation models for remote sensing images adopt an encoder-decoder structure, where image features are compressed in the spatial dimension during the encoding process. However, due to the inherently fuzzy boundaries of remote sensing images, it is challenging to fully recover the shallow detail information, especially edge information, during decoding. This limitation prevents the segmentation results from accurately fitting the actual shapes of objects, resulting in blurry segmentation boundaries. Furthermore, remote sensing images often exhibit significant variations in feature scale. Single-scale analysis methods struggle to accurately capture features across different scales, making the model prone to omissions or misdetections in complex scenes. These challenges ultimately limit the model’s performance in practical applications.

To address the issue of improving semantic segmentation accuracy for remote sensing images with blurry object edges and varying scales, a boundary-enhanced multi-scale semantic segmentation model for remote sensing images, BEMS-UNetFormer, is proposed. The main contributions are summarized as follows:

- This paper proposes a boundary-enhanced semantic segmentation network for multi-scale remote sensing images, called BEMS-UNetFormer. The network incorporates the Gated Channel Transformation (GCT) into the Boundary-Aware Attention Module (BAM) to extract shallow contour information, effectively enhancing important feature channels and suppressing noisy boundaries. Additionally, inspired by the Attention Mechanism, the Boundary-Guided Fusion Module (BFM) is improved to strengthen the network’s ability to extract and integrate edge features.
- The proposed Multi-Scale Cascade Atrous Convolution Module (MSC-ASPP), which is capable of extracting features at different scales to obtain richer contextual information and effectively integrate global and local information. Compared with traditional methods, this module shows stronger adaptability in dealing with complex scenes and fine objects.
- The effectiveness of BEMS-UnetFormer was validated on two benchmark datasets, ISRPS Potsdam and Vaihingen, achieving 86.12% and 83.10% MIoU through quantitative and qualitative experiments, respectively.

## Related works

### UNetFormer

UNetFormer is an efficient semantic segmentation model that combines the multi-scale feature extraction capabilities of UNet with the global context modeling of Transformer. The model consists of two main parts: a CNN-based encoder and a Transformer-based decoder. The encoder employs the lightweight ResNet18 backbone^23^ as a feature extractor. ResNet18 is a classic CNN with fewer parameters and lower computational complexity, while maintaining strong feature extraction capabilities. The decoder incorporates three Global-Local Attention Blocks (GLTB) to capture both global and local information. GLTB enables the model to focus on the entire image and local details simultaneously, thereby enhancing the model’s ability to capture comprehensive semantic information. Additionally, the decoder includes a Feature Refinement Head (FRH) to refine both channel-wise and spatial feature representations. Although UNetFormer can effectively capture semantic information in images, it struggles to precisely align with object boundaries, resulting in imprecise and blurred segmentation boundaries. The UNet structure used in the model naturally facilitates multi-scale feature fusion. However, further refinement of the multi-scale fusion strategies is needed to better capture fine-grained details and contextual information in images.

### Boundary detection

In the context of boundary optimization in remote sensing image segmentation, Luo et al.^24^ employed the traditional Sobel edge detection operator to extract the edges of the saliency map. Wei et al.^25^ introduced a polygon regularization post-processing method after FCNs to optimize the initial results of buildings extracted by the semantic segmentation network. Guo et al.^26^ developed a coarse-to-fine boundary refinement network (CBRNet) to accurately extract buildings from high-resolution remote sensing images. Cheng et al.^27^ designed a multi-task network for segmentation and edge detection, providing complementary information to enhance gradient learning of the entire model. To enhance context integration, Chong et al.^28^ proposed an information exchange mechanism (IEM) based on a dual-stream network to refine the boundaries of small-scale objects. Zheng et al.^29^ applied a boundary supervision auxiliary module to restore boundary contours, mitigating blurring effects when constructing contextual information. Wang et al.^30^ designed a local-aware attention module to optimize the edge information of target objects by adaptively adjusting the weight information of local feature points. Ni et al.^31^ proposed an edge-guided network (EIGNet) for semantic segmentation of high-resolution remote sensing images, incorporating a directional convolution module to construct spatial detail branches for accurate edge and spatial detail information. Cui et al.^32^ proposed a global context-dependent perceptual network (GCDNet) and designed an edge-aware optimization module, which directly computes the edge loss on the edge regions to optimize edge details and achieve more accurate target edge segmentation results. Zhou et al.^33^ proposed a lightweight semantic segmentation network, BSCNet, which utilizes an Extremely Lightweight Pyramid Pooling Module (ELPPM) to capture multi-scale semantic context and introduces a Boundary-Assisted Fusion Module (BAFM) to enhance boundary performance by optimizing low-level convolutional features through boundary prediction. Liu et al.^34^ developed a Cross-Fusion Model (CF2N) for fine-grained detail reconstruction and fusion in remote sensing images. This model integrates a Frequency-Domain-Driven Detail Reconstruction strategy (FD2R) and a Frequency-Spectrum Cross-Fusion Module (FSCF), achieving high-fidelity fusion results by adaptively merging frequency details and facilitating high-frequency interactions.

### Multi-scale aggregation

Ma et al.^35^ proposed SACANet, which effectively tackles the challenges of complex backgrounds and large intraclass variance in remote sensing images through a local-global class attention mechanism. Li et al.^36^ proposed a Contextual Semantic Refinement Network, which integrates local segmentation results with their contextual semantics, thereby reducing boundary artifacts and optimizing mask contours during the generation of the final high-resolution mask. Huang et al.^37^ proposed a multi-scale fusion semantic segmentation network DRCNet based on the attention mechanism, which significantly enhances the segmentation accuracy of targets of different sizes through cross-layer interaction of multi-scale feature maps. Zhang et al.^38^ proposed an adaptive multi-scale branch network, SANNet, to segment targets of different sizes, by merging the results of each branch to achieve superior performance. Wu et al.^39^ employed a channel and spatial attention fusion module (AFM) to adaptively fuse deep semantic features and shallow detail features, achieving the extraction and adaptive fusion of local features and global contextual information. FTransUNet proposed by Ma et al.^40^ enhanced the recognition of key semantic regions by merging the surface details and deeper background information of an image at multiple levels. Ma et al.^41^ provided an in-depth review of the pre-training process of Transformer and proposed the SatMAE++. The model employs a multi-scale pre-training strategy and incorporates a convolution-based up-sampling module to reconstruct higher resolution images, enabling effective multi-scale information integration. Wang et al.^42^ introduced an efficient remote attention module for capturing large-scale contextual information or long-term dependencies, and designed a novel multi-scale local attention module for capturing detailed local information.

## Methods

**Fig. 1:**
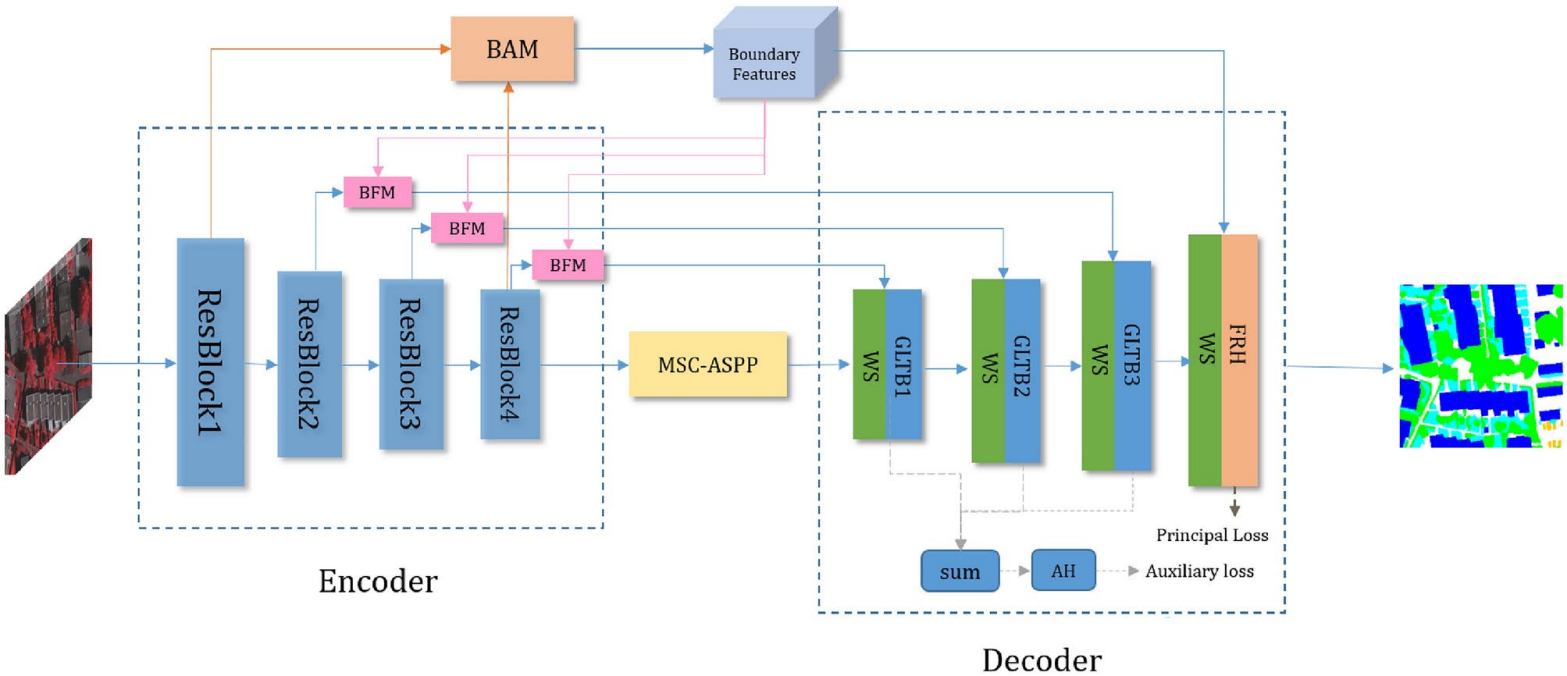
An overview of the BEMS-UNetFormer.

The challenges of fuzzy target edges and variable scales in high-resolution remote sensing images hinder the improvement of semantic segmentation accuracy. To tackle these challenges, this work proposes a Boundary-Enhanced Multi-Scale Semantic Segmentation Network (BEMS-UNetFormer) based on UNetFormer. Figure 1 illustrates the overview of the proposed framework. Specifically, an improved Boundary Awareness Module (BAM) is designed to extract edge information of the target from high-level semantic features and low-level features containing edge details, effectively mitigating blurred edges in the final segmentation. Subsequently, the improved Boundary-Guided Fusion Module (BFM) integrates the edge information from BAM with multi-level backbone features at each level, facilitating its integration into subsequent decoding processes, thereby better preserving boundary details and enhancing boundary representation. Lastly, the Multi-Scale Cascaded Atrous Spatial Pyramid Pooling (MSC-ASPP) is employed to enhance feature fusion within the network, improving segmentation accuracy. The MSC-ASPP module extracts multi-scale features through atrous convolution operations, enhancing the network’s ability to segment targets of varying scales. Additionally, the MSC-ASPP module gradually fuses multi-scale features through a cascaded approach, further improving segmentation accuracy.

### Boundary awareness module (BAM)

In the process of feature extraction, shallow convolutional layers are primarily responsible for capturing fine-grained information, such as edges, contours, textures, and other low-level details, while deep convolutional layers focus on extracting abstract semantic information related to categories. Based on this characteristic, this work introduces a Boundary-Aware Module (BAM)^43^ to extract edge information from low-level features. However, while capturing a large amount of detailed features, it also introduces considerable noise. Inspired by the use of gating mechanisms in natural language processing tasks to control the transmission state and address noise issues, this work integrates a Gated Channel Transformation (GCT) module into the BAM to filter noise from low-level features, thereby enhancing key feature channels and effectively suppressing noisy boundaries.

The GCT captures the global features of each channel by embedding global context information, thereby mitigating local semantic ambiguities and enabling better access to generic attributes for shallow features. Through normalization operations, the GCT establishes competition between channels, enhancing channels with stronger responses while suppressing channels with weaker feedback. This normalization method establishes competition between neurons or channels, thereby enhancing model performance. The GCT facilitates channel selection through gating weights and biases. When the gating weights of a channel are positively activated, GCT enhances the channel’s competitive behavior; when the gating weights are negatively activated, GCT promotes the channel’s cooperative behavior. This mechanism significantly enhances critical feature channels and suppresses noisy boundaries.

**Fig. 2:**
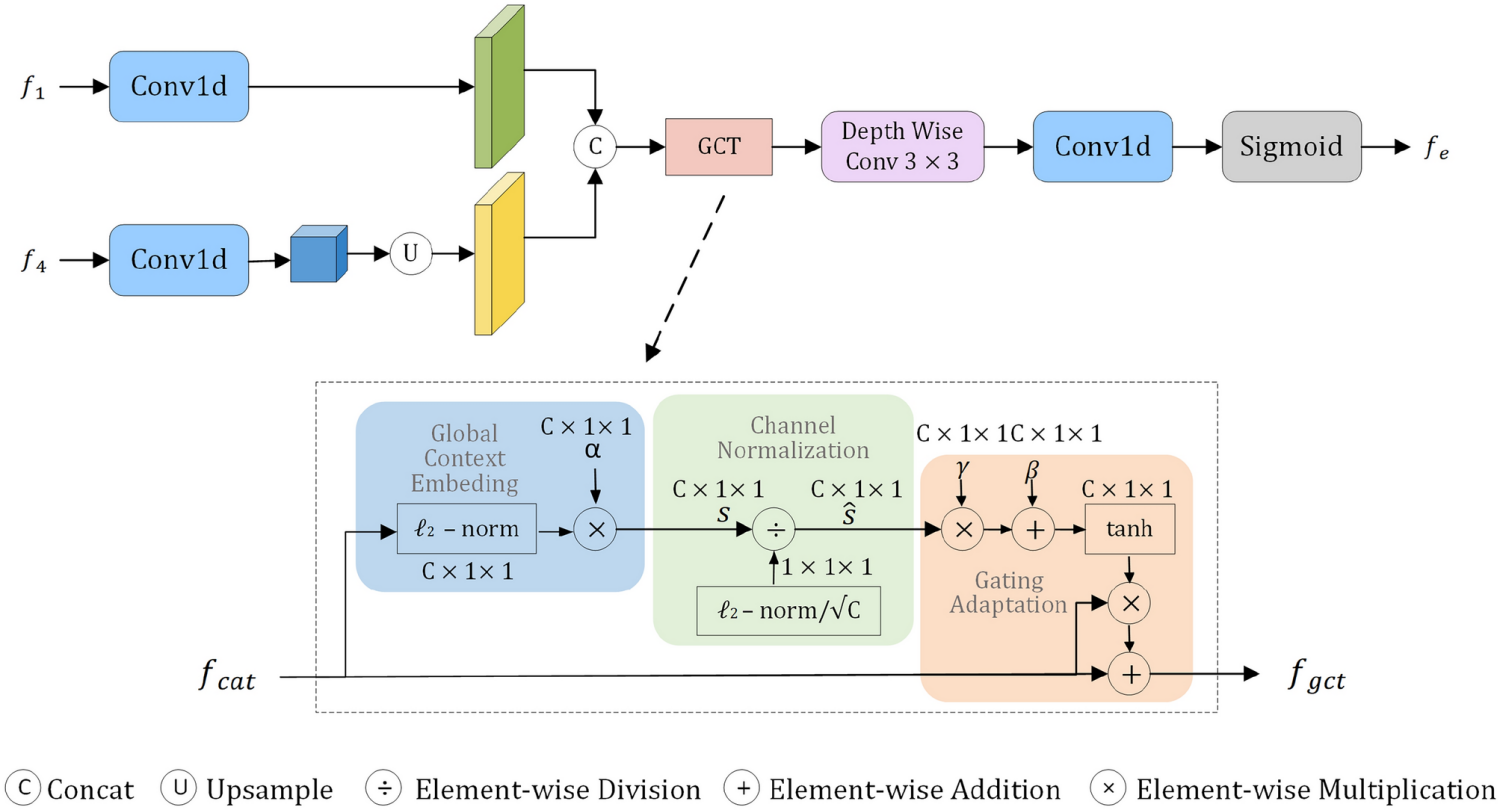
Improved boundary awareness module (BAM).

The structure of the improved BAM is shown in Figure 2. Firstly, the input of this module comes from the output feature maps of the first and fourth layers (Res1 and Res4) of the encoder. By using 1x1 convolution, the channel numbers of the low-level feature block $f_{1}$ and the high-level feature block $f_{4}$ are adjusted, and $f_{1}$ is upsampled to match the size of $f_{4}$. Then, the two are concatenated and fed into the GCT, which adaptively adjusts the channel weights to enhance important feature channels, suppress noisy boundaries, and enhance contextual relevance. The features are then fused through two 3x3 convolution operations. Finally, edge features are obtained through a 1x1 convolution and a sigmoid function. In this manner, the BAM extracts edge-related features from various levels of the encoder and utilizes the GCT to strengthen critical features, leading to more precise boundary predictions and thereby enhancing the accuracy of remote sensing image segmentation tasks. The above process can be represented by equations (1) to (5).

1 $$\begin{aligned} f'_{1}&=F_{conv1}(f_1), f'_{4}=F_{conv4}(f_4) \end{aligned}$$

2 $$\begin{aligned} f_{cat}&=f'_{1}\otimes f'_{4} \end{aligned}$$

3 $$\begin{aligned} f_{gct}&=GCT(f_{cat}) \end{aligned}$$

4 $$\begin{aligned} f_{fuse}&=F_{conv3}(F_{conv3(f_{gct})}) \end{aligned}$$

5 $$\begin{aligned} f_{e}&=\sigma (F_{conv1}(f_{fuse})) \end{aligned}$$

The symbol $\otimes$ represents element-wise addition, GCT() denotes the Gated Channel Transformation unit formula, $F_{conv1}$ is a 1$\times$1 convolution, $F_{conv3}$ is a 3$\times$3 convolution.

### Boundary-guided fusion module (BFM)

This work proposes an improved Boundary-Guided Fusion Module (BFM), which facilitates more effective fusion of edge priors and backbone features through the use of Depthwise Separable Convolutions^44^, Coordinate Attention^12^, and enhanced spatial information encoding strategies, thereby significantly enhancing the model’s performance in edge detail feature extraction.

This work employs 3$\times$3 Depthwise Separable Convolutions to replace standard 3$\times$3 convolutions. Depthwise Separable Convolutions decompose the convolution into depthwise convolution and pointwise convolution, significantly reducing both computational complexity and the number of parameters while maintaining feature extraction capabilities. To enhance the fusion of edge priors and backbone features, the BFM incorporates Coordinate Attention. This mechanism decomposes channel attention into two one-dimensional feature encoding processes, enabling it to aggregate features along both horizontal and vertical directions. This approach not only preserves the inter-channel dependencies but also incorporates spatial location information, enabling the model to capture the location of the target region more accurately. Specifically, Coordinate Attention achieves its functionality in two steps: firstly, it conducts global information aggregation along one direction (horizontal or vertical), and then performs the same operation in the other direction. This bidirectional aggregation process allows the model to capture richer spatial information and long-range dependencies. To further enhance the BFM’s edge feature extraction capability, this work also proposes an improved spatial information encoding strategy. This strategy introduces additional spatial encoding layers to enhance the encoding and representation of input features in the spatial dimension, enabling the model to better understand the spatial relationships between different positions in the image. This explicit encoding of spatial information enables the model to more accurately locate edge positions and retain more edge detail information when integrating edge priors and backbone features.

**Fig. 3:**
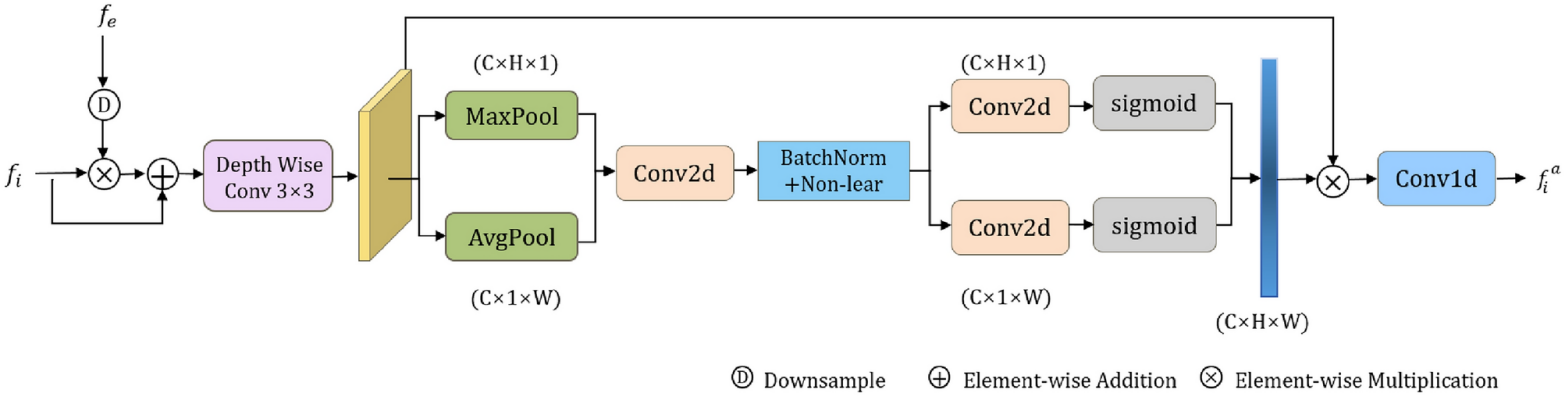
Improved boundary-guided aggregation module (BFM).

As shown in Figure 3, the input to the BFM module consists of edge features $f_i (i \in \left\{ 2, 3, 4 \right\} )$ and semantic features $f_e$. Firstly, the initial fusion feature $f^e_i$ is obtained by jump-joining and 3$\times$3 Depthwise Separable Convolutions after element-level multiplication of the edge features. Secondly, the feature vectors perceived in both directions are obtained by global average pooling of the feature map $f^e_i$ in horizontal and vertical directions, respectively. This enables the model to focus on key features independently in both horizontal and vertical directions, thus capturing edge details more accurately. Then, the concatenated direction-aware feature vectors are encoded through one-dimensional convolutions, batch normalization, and non-linear activation functions to capture spatial information. Finally, after one-dimensional convolution transformation and sigmoid normalization, two attention maps corresponding to the horizontal and vertical directions are generated, and these attention maps are multiplied by $f^e_i$ to obtain the output feature map. The above process can be represented by equations (6) to (10).

6 $$\begin{aligned} f^e_i&=F_{DSConv}(f_i\otimes D(f_e))\otimes f_i \end{aligned}$$

7 $$\begin{aligned} f^h_i&=1/H {\textstyle \sum _{i=1}^{H}}f^e_i, f^w_i=1/W {\textstyle \sum _{i=1}^{W}}f^e_i \end{aligned}$$

8 $$\begin{aligned} G_h&=\sigma (F_{conv1}(F_1;w)),G_w=\sigma (F_{conv1}(F_{w+1};h+w)) \end{aligned}$$

9 $$\begin{aligned} f_{out}&=\sigma (F_{conv1}(f_{fuse})) \end{aligned}$$

10 $$\begin{aligned} F&=(G_h\otimes G_w)\odot f^e_i \end{aligned}$$

### Multi-scale cascaded atrous spatial pyramid pooling (MSC-ASPP)

**Fig. 4:**
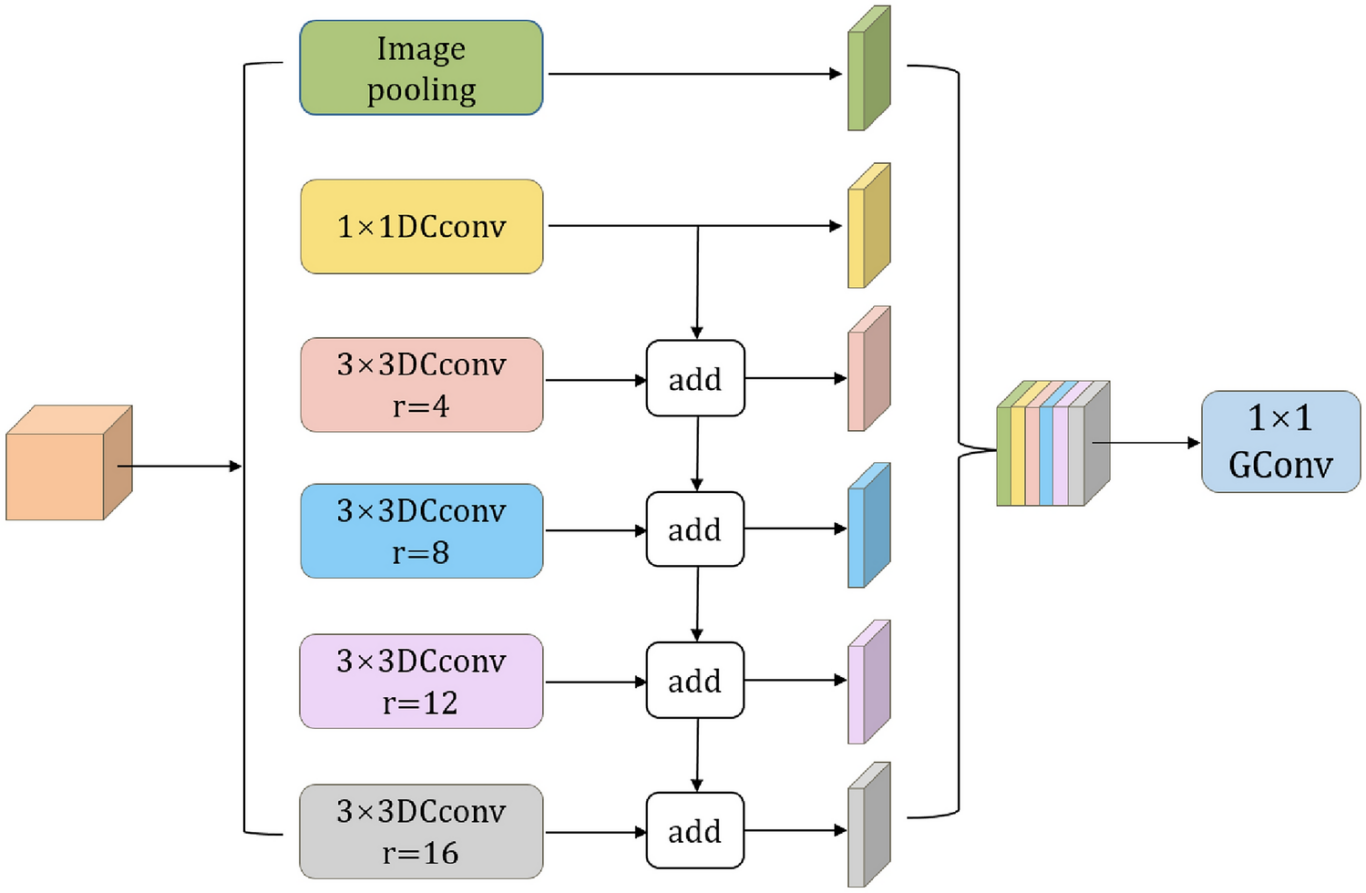
Multi-scale cascade atrous convolution (MSC-ASPP).

ASPP, a module in DeepLabv3+, is designed for extracting multi-scale features and typically employs dilation rates of 6, 12, and 18. However, as the backbone network extracts features, the resolution of the feature maps gradually decreases, rendering the combination of 6, 12, and 18 less effective for extracting features from multi-resolution feature maps. Additionally, the lack of smaller atrous rates leads to suboptimal performance in segmenting small targets, weakening the model’s ability to handle segmentation tasks of varying sizes. To enhance the extraction of features from multi-resolution feature maps and improve the segmentation capability for targets of different sizes, this work modifies the atrous convolution rates to 4, 8, 12, and 16. To minimize computational costs, standard convolutions are replaced with Depthwise Separable Convolutions. To further enhance feature utilization efficiency, multi-level fused feature information is obtained by pooling the input features and concatenating them with the multi-scale features. The improved ASPP is referred to as the Multi-Scale Cascaded Atrous Spatial Pyramid Pooling (MSC-ASPP), as shown in Figure 4.

### Loss function

In the original UNetFormer, an additional auxiliary feature head was constructed during training to optimize features, using both the main loss and auxiliary loss to train the entire network. The main loss $\mathscr {L}_{\text{ p }}$ is a combination of DICE loss $\mathscr {L}_{\text{ dice } }$ and cross-entropy loss, and the auxiliary loss is further multiplied by a factor $\alpha$. However, the two losses in the main loss do not have balancing parameters, and the auxiliary loss factor is set to a default value of 0.4, which is not learnable. This means that during training, these weights and factors remain constant and do not adaptively adjust based on the characteristics of the data. To adaptively balance the two losses in the main loss or adjust the auxiliary loss factor $\alpha$, balancing parameters are introduced for the DICE loss $\mathscr {L}_{\text{ dice } }$ and cross-entropy loss $\mathscr {L}_{\text{ ce } }$ in the main loss to control their relative contributions to the total loss. The auxiliary loss factor $\alpha$ is set as a trainable variable and is updated during training through gradient descent. The above process can be represented by equations (11) to (14).

11 $$\begin{aligned} \mathscr {L}_{c e}&=-\frac{1}{N} \sum _{n=1}^N \sum _{k=1}^K y_k^{(n)} \log \hat{y}_k^{(n)} \end{aligned}$$

12 $$\begin{aligned} \mathscr {L}_{\text{ dice } }&=1-\frac{2}{N} \sum _{n=1}^N \sum _{k=1}^K \frac{\hat{y}_k^{(n)} y_k^{(n)}}{\hat{y}_k^{(n)}+y_k^{(n)}} \end{aligned}$$

13 $$\begin{aligned} \mathscr {L}_p&=\beta \mathscr {L}_{c e}+(1-\beta ) \mathscr {L}_{\text {dice }} \end{aligned}$$

14 $$\begin{aligned} \mathscr {L}&=\mathscr {L}_p+\alpha \times \mathscr {L}_{\textrm{aux}} \end{aligned}$$

## Experiments and results

###  Experimental dataset

In this work, the Vaihingen dataset and Potsdam dataset published by ISPRS were selected for the experiments.

The Potsdam dataset highlights the diverse urban landscape of Potsdam, Germany, encompassing 38 distinct urban areas that span categories ranging from dense residential neighborhoods to industrial zones. Utilizing remotely sensed imagery with an impressive spatial resolution of 0.05 meters, this dataset ensures highly detailed feature recognition. Each image measures 6000 $\times$ 6000 pixels, a scale that not only challenges the efficiency of processing algorithms but also demands their capability to capture complex spatial relationships within large scenes. The dataset is meticulously annotated with 6 land cover classes: Impervious surfaces(Street), Building, Low vegetation(LowVeg), Tree, Car, and Background/Clutter.

In contrast, the Vaihingen dataset originates from digital aerial photography of Vaihingen, Germany, and consists of 33 remotely sensed images with distinct dimensions and predefined semantic labels. These high-resolution images, at 0.09 meters per pixel, are well-suited for detailed analyses. Their sizes vary, ranging from 1000 to 4000 pixels in both width and height. Captured across the near-infrared, red, and green spectral bands, the images offer a multi-spectral perspective of the urban environment. The dataset includes 6 categories: Impervious surfaces (e.g., Street, RGB: 255, 255, 255), LowVeg (RGB: 0, 255, 255), Building (RGB: 0, 0, 255), Tree (RGB: 0, 255, 255), Car (RGB: 255, 255, 0), and Background(RGB: 255, 0, 0).

**Fig. 5:**
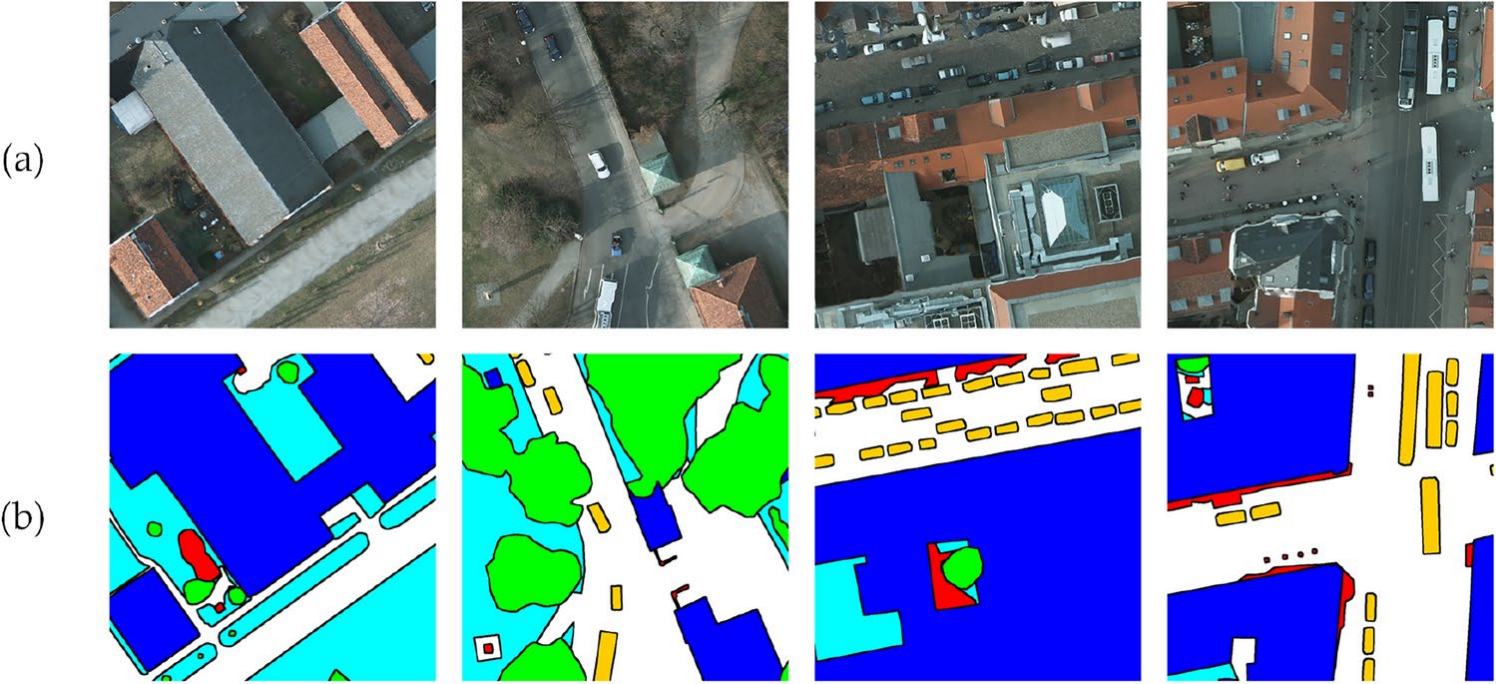
Example plot of Potsdam dataset: (**a**) Original, (**b**) Label.

**Fig. 6:**
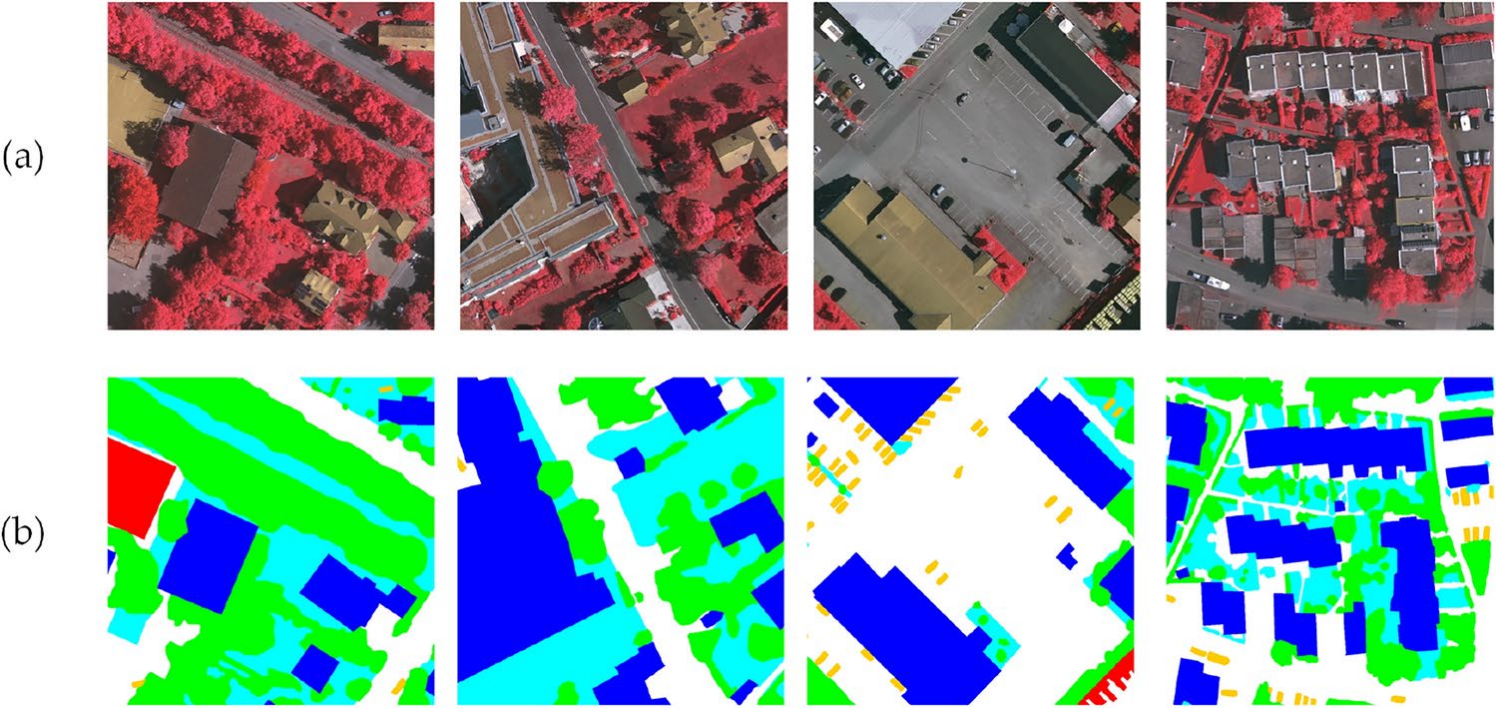
Example plot of Vaihingen dataset: (**a**) Original, (**b**) Label.

During the experiments, the training and test sets were divided according to the ratio of 8:2. For the Potsdam dataset, 8 images from the Potsdam dataset (numbered 2-10, 3-11, 4-15, 5-13, 6-11, 6-15, 7-9 and 7-12) were selected as the test set, while the remaining 30 images were used as the training set. Similarly, for the Vaihingen dataset, 27 images constituted the training set, with the remaining 6 images (numbered 3,9,13,15,20 and 28) forming the test set. Examples of processed datasets are shown in Figs. 5 and 6. Since high-resolution remote sensing images are generally large, preprocessing operations such as cropping were performed to resize the images to 1024 $\times$ 1024 pixels before semantic segmentation. After cropping, the Potsdam dataset contained 2792 training set images and 696 test set images, while the Vaihingen dataset contained 1168 training set images and 290 test set images.

###  Experimental environment

The experiments were carried out within a Linux environment, with the detailed system specifications outlined in Table 1 below. During the network training phase, we set the learning rate to $6\times 10^{-5}$ specifically for the backbone network. A batch size of 8 was employed, and the Lookahead optimizer was chosen to optimize the training process. The model was trained for 45 epochs on the Potsdam dataset and 225 epochs on the Vaihingen dataset.

**Table 1:** Experimental environment parameters.

**Experimental Platform**	**Run Parameters**
Operating System	Ubuntu Server 20.04
Processor	Intel(R) Xeon(R) CPU E5-2630 v4
Memory	24 GB
Disk	500 GB
Graphics Card	Tesla P40
Framework	Pytorch
Acceleration Library	CUDA 11.8

###  Evaluation metrics

To quantitatively assess the performance of the network in semantic segmentation, four evaluation metrics were defined: MIoU, F1, MF1, and OA. These metrics are computed using formulas (15) to (18). Here, TP, FP, TN, and FN represent true positives, false positives, true negatives, and false negatives, respectively, while k denotes a specific category. MIoU measures the mean IoU across categories, providing a balanced evaluation of performance. F1 and MF1 consider both precision and recall, with MF1 averaging the F1 scores across all categories. OA, on the other hand, indicates the proportion of correctly predicted pixels overall, offering a general overview of accuracy.

15 $$\begin{aligned} OA &= \frac{TP + TN}{TP + FP + TN + FN} \end{aligned}$$

16 $$\begin{aligned} MIoU&= \frac{1}{N}{\textstyle \sum _{k-1}^{N}} \frac{TP_k}{TP_k+FP_k+FN_k} \end{aligned}$$

17 $$\begin{aligned} F1&=2\times \frac{precision\times recall }{precision+ recall } \end{aligned}$$

18 $$MF1 = \frac{1}{N} \sum_{k=1}^{N} F1_k$$

###  Comparative experiments

To evaluate the effectiveness of the proposed model, BEMS-UNetFormer, it was compared with several mainstream models in the field of remote sensing image segmentation, including SegFormer^45^, UNetMamba, LOGCAN ++^46^, SFA-Net^47^ and UNetFormer. All models were trained and tested under identical conditions on a unified hardware and software platform to ensure fairness, data quality, and reliable comparisons.

#### Experimental results for potsdam data

The comparison results of segmentation performance on the Potsdam dataset are shown in Table 2. In terms of overall performance, the proposed method achieves MIoU, OA, and MF1 scores of 91.56%, 86.12%, and 92.43%, respectively, representing improvements of 1.38%, 1.02%, and 0.80% over the baseline model. For small-scale targets like “Car,” BEMS-UNetFormer achieves IoU and F1 scores of 91.20% and 95.57%, respectively, second only to LOGCAN++, highlighting its robustness in handling fine-scale segmentation tasks. Moreover, the proposed method demonstrates superior performance for densely distributed targets such as “LowVeg,” attaining the highest IoU and F1 scores of 78.46% and 87.93%, respectively. Compared with other mainstream models, BEMS-UNetFormer exhibits more balanced and precise recognition capabilities, excelling in both small-scale and densely distributed target segmentation.

**Table 2:** Comparative experiments on the potsdam dataset.

**Model**	**Street**		**Building**		**LowVeg**		**Tree**		**Car**		**OA**	**MIoU**	**MF1**
	**IoU**	**F1**	**IoU**	**F1**	**IoU**	**F1**	**IoU**	**F1**	**IoU**	**F1**			
SegFormer	38.02	55.09	76.20	86.49	75.47	86.02	77.89	87.57	68.93	81.61	86.29	70.99	81.87
UnetMamba	65.23	78.96	90.76	95.16	77.28	87.18	79.69	88.70	90.91	95.24	91.09	85.59	92.13
LOGCAN++	42.92	60.06	**92.89**	**96.31**	77.19	87.13	**80.31**	**89.08**	**91.65**	**95.65**	90.80	85.77	92.22
SFA-Net	69.27	80.02	91.59	95.61	78.04	87.63	79.58	88.63	90.90	95.23	91.51	**86.12**	92.42
UNetFormer	63.84	77.94	88.64	93.97	76.83	86.90	78.99	88.26	91.19	95.39	90.54	84.74	91.63
Ours	**69.36**	**81.91**	91.58	94.57	**78.46**	**87.93**	79.34	88.48	91.20	95.57	**91.56**	**86.12**	**92.43**

**Fig. 7:**
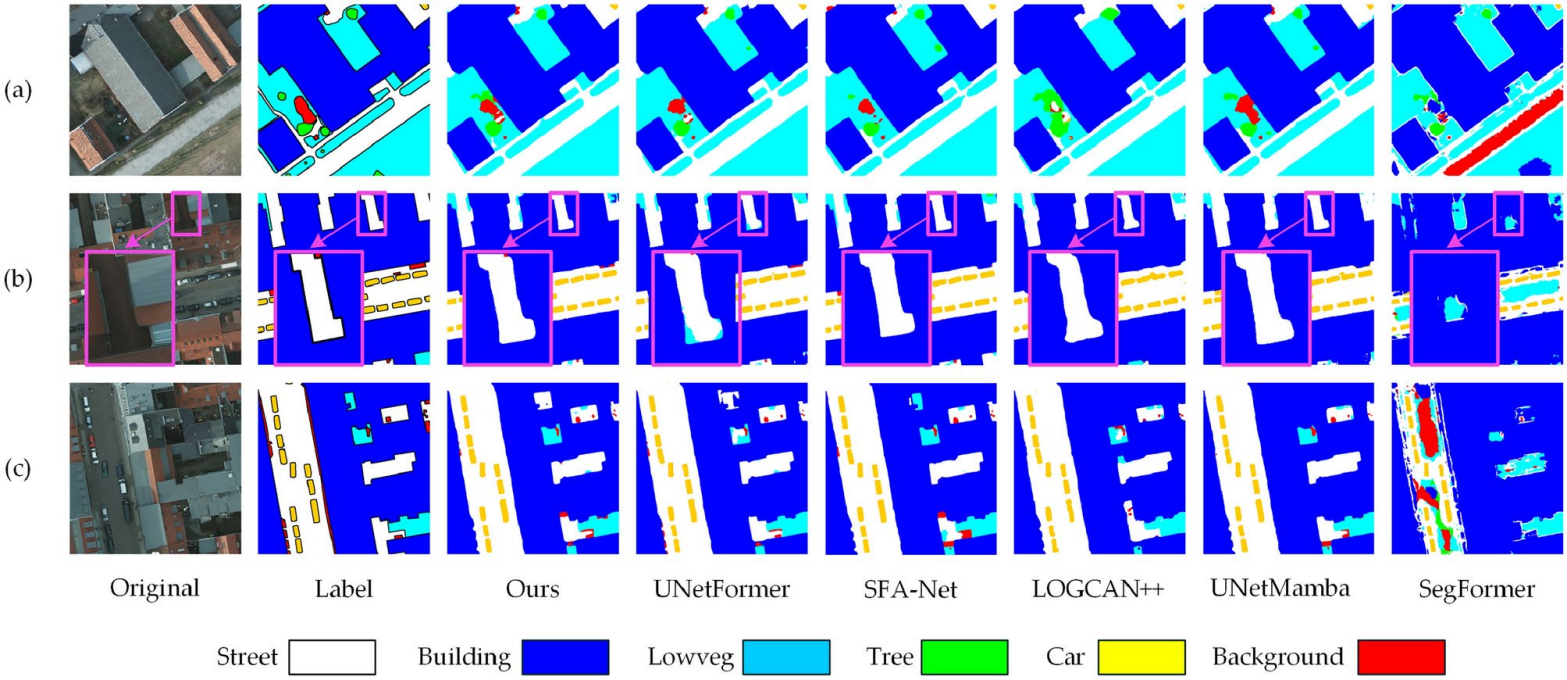
Comparison of segmentation results on the Potsdam dataset with general segmentation models: (**a**), (**b**) and (**c**) represent the segmentation results of three different scenarios.

A comparison of BEMS-UNetFormer and the mainstream segmentation network’s segmentation results on the Potsdam dataset is shown in Figure 7. Observing Figure 7(a), it is evident that the model occasionally misdetects boundaries when elements are densely packed, which places high demands on the model’s ability to capture both local and global semantic information. The segmentation map generated by the BEMS-UNetFormer method closely resembles the labeled map, with relatively clear segmentation boundaries. Additionally, LOGCAN++ sometimes misclassifies the background as trees, whereas the proposed method accurately recognizes it as background. Figure 7(b) shows that the model proposed in this paper is more accurate in segmenting long targets such as “Building”, “Street”, and “Car”. In the locally enlarged area, the boundary segmented by the proposed model is closest to the labeled map. In contrast, the UNetFormer model has incorrectly recognized some areas of “LowVeg” along its boundaries, while the SegFormer is unable to segment the shape of the boundary. Figure 7(c) shows that the overall recognition performance of the proposed model is noticeably better. However, the UNetFormer and LOGCAN++ models confuse “Building” with “Street”, and the SFA-Net misclassifies some areas of “LowVeg” as “Background”.

**Fig. 8:**
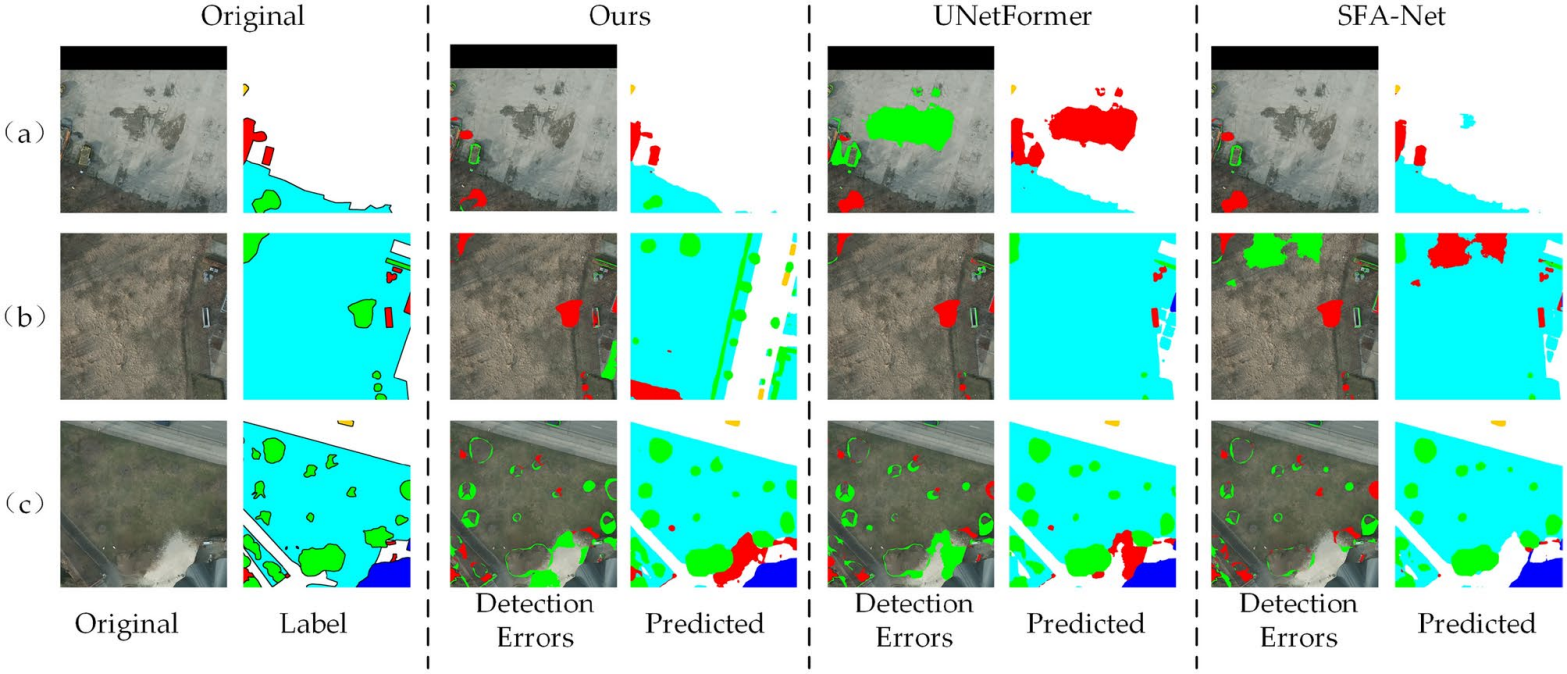
Visualization results of missed and misdetected pixels on the potsdam dataset.

In this paper, different colors are employed to visualize missed and misdetected pixels, with the visualization results for the Potsdam dataset illustrated in Figure 8. To evaluate the model’s performance, the proposed BEMS-UNetFormer model is compared with the base model UNetFormer and the frontier model SFA-Net. In the visualization, “Detection Errors” highlights the distribution of missed and misdetected pixels, where red areas represent missed pixels, green areas indicate misdetected pixels, and “Predicted” displays the prediction results of each model. As shown in the center region of Figure 8(a), the number of missed and misdetected pixels produced by the proposed model is significantly lower compared to the other models. By contrast, the UNetFormer model shows large-scale misdetections in this region, represented by a prominent red background. In the upper section of Figure 8(b), the SFA-Net incorrectly classifies parts of low vegetation as red background, whereas both the proposed model and the base model successfully identify this region. Figure 8(c) indicate that although the proposed model outperforms the comparison model in overall performance, there are still a small number of omissions and misdetections in the tree detection task, which is similar to the limitations of the comparison models. Collectively, these findings underscore the advantages of the proposed model in improving boundary clarity and recognition accuracy in complex environments.

#### Experimental results for vaihingen data

The comparison results of segmentation performance on the Vaihingen dataset are presented in Table 3. The results demonstrate that BEMS-UNetFormer achieves superior performance across all comprehensive evaluated metrics. Specifically, the proposed model outperforms the baseline model, with MIoU, OA, and MF1 metrics increasing by 1.79%, 0.87%, and 1.14%, respectively. For the street category, which exhibits large variations in geometry and texture, the model proposed in this paper achieves an IoU of 90.40% and an F1 score of 94.96%, representing improvements of 2.10% and 1.18%, respectively, compared to the baseline model. In the segmentation task of two confusable categories, “LowVeg” and “Tree”, the IoU and F1 score for low vegetation reach the highest values of 74.46% and 85.36%, respectively. Furthermore, in the “Building”, the IoU and F1 score of the proposed model reach 92.30% and 96.00%, respectively, which are the highest among all compared models. These results underline the effectiveness of BEMS-UNetFormer in segmenting categories with complex spatial and structural features, demonstrating its capability to achieve precise and reliable performance.

**Table 3:** Comparative experiments on the vaihingen dataset.

**Model**	**Street**		**Building**		**LowVeg**		**Tree**		**Car**		**OA**	**MIoU**	**MF1**
	**IoU**	**F1**	**IoU**	**F1**	**IoU**	**F1**	**IoU**	**F1**	**IoU**	**F1**			
SegFormer	21.22	35.01	90.47	95.00	71.32	83.26	80.37	89.12	**80.37**	**89.12**	89.59	69.21	79.10
UnetMamba	82.65	90.50	91.26	95.43	72.54	84.08	80.37	89.11	73.56	84.77	91.42	81.63	89.67
LOGCAN++	83.98	91.29	91.89	95.77	72.57	84.10	80.15	88.98	75.32	85.92	91.47	82.06	89.94
SFA-Net	85.18	92.00	92.16	95.92	74.40	85.32	**81.29**	**89.68**	75.60	86.68	92.04	83.07	90.58
UNetFormer	88.30	93.78	91.82	95.73	71.91	83.66	79.26	88.43	72.99	84.39	91.22	81.31	89.45
Ours	**90.40**	**94.96**	**92.30**	**96.00**	**74.46**	**85.36**	81.15	86.50	76.22	86.50	**92.09**	**83.10**	**90.59**

**Fig. 9:**
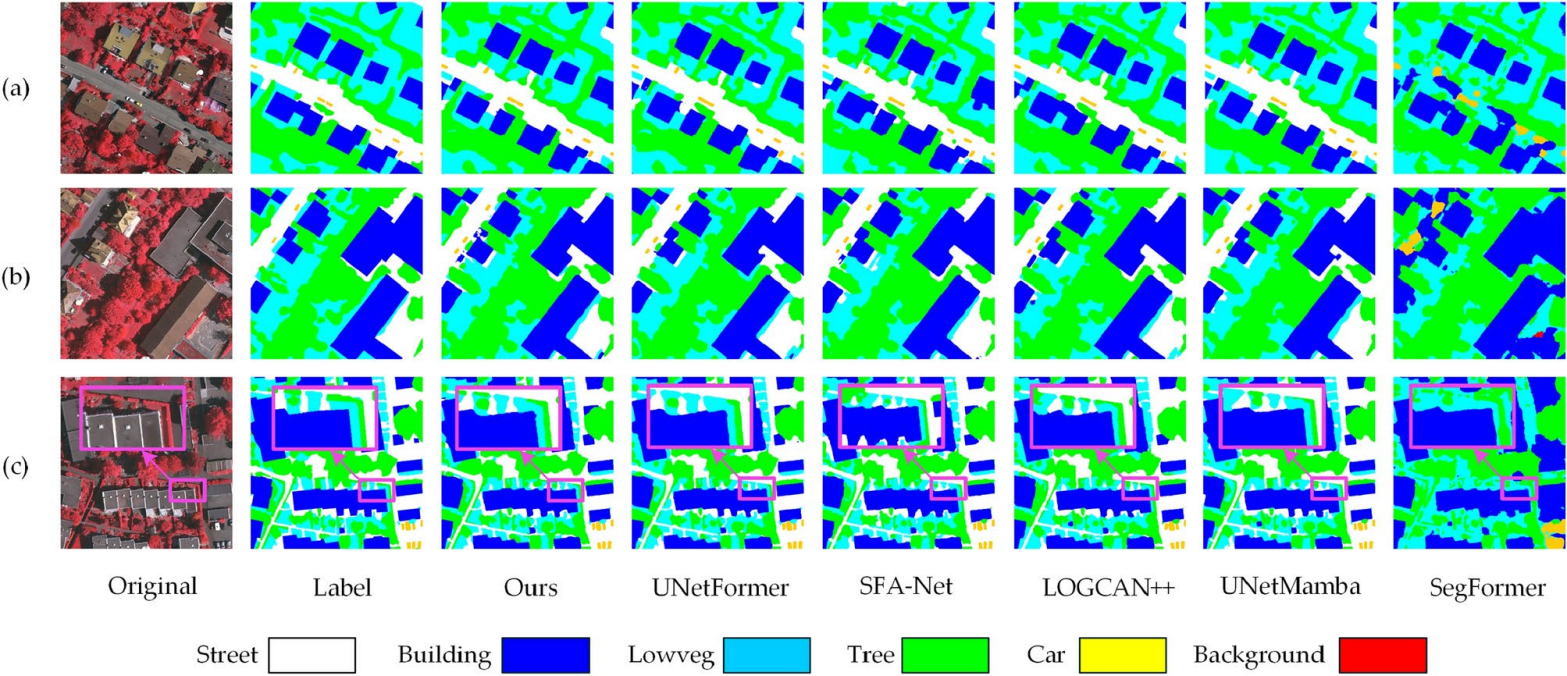
Comparison of segmentation results on the Vaihingen dataset with general segmentation models: (**a**), (**b**) and (**c**) represent the segmentation results of three different scenarios.

A comparison of the segmentation results of BEMS-UNetFormer with mainstream segmentation networks on the Vaihingen dataset is shown in Figure 9. In Figure 9(a), the proposed model outperforms other models in segmenting the contours of cars and buildings. In Figure 9(b), when processing densely packed and visually similar elements, most models tend to misclassify trees as low vegetation. In contrast, the proposed model demonstrates significantly higher accuracy in distinguishing these categories. As shown in the magnified region of Figure 9(c), the building boundaries segmented by the proposed model are the closest to the labeled map. In comparison, SFA-Net and LOGCAN++ exhibit noticeable jagged edges along the boundaries. Overall, compared to other models, BEMS-UNetFormer achieves substantial improvements in segmenting small-sized targets and defining target boundaries. The experimental results confirm the effectiveness of the proposed enhancements, leading to a significant increase in segmentation precision and accuracy, particularly in complex scenarios.

**Fig. 10:**
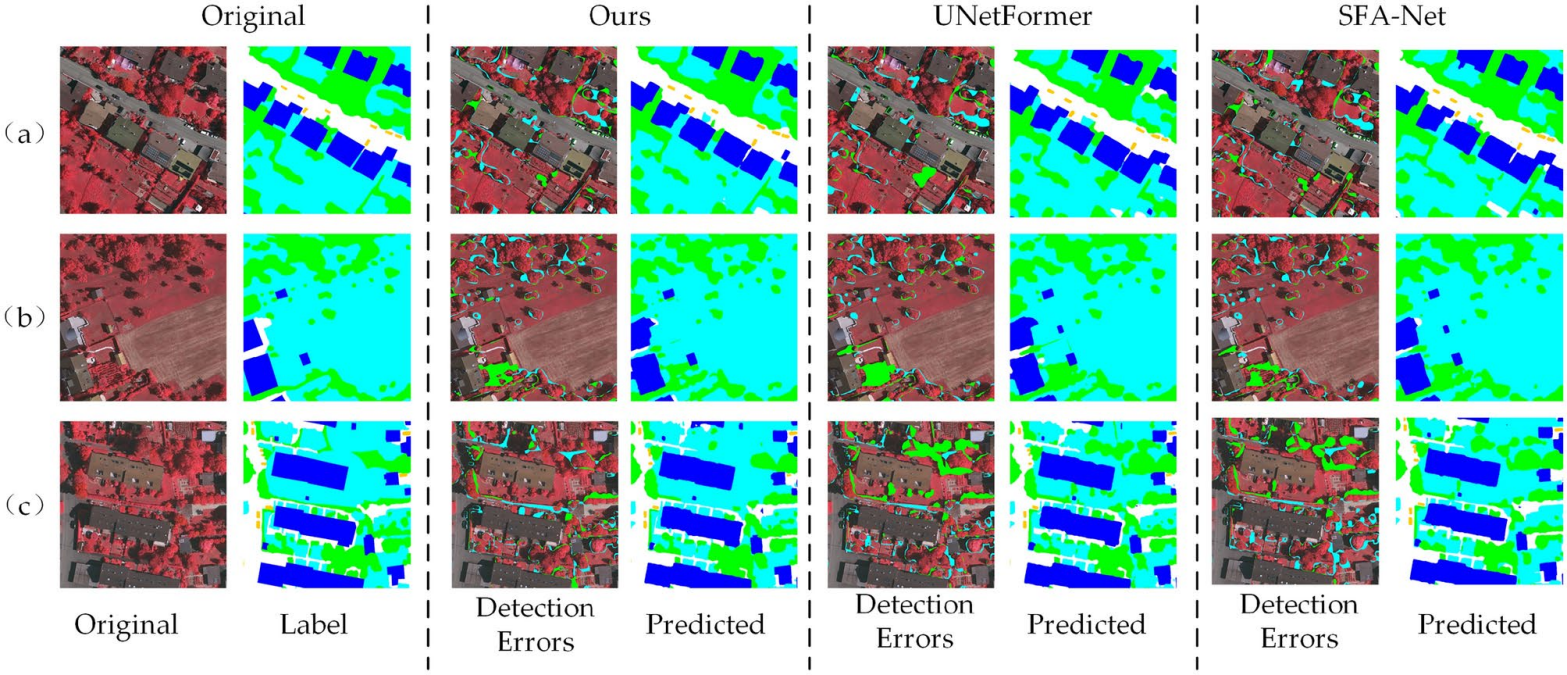
Visualization results of missed and misdetected pixels on the vaihingen dataset.

The results of the visualization of missed and misdetected pixels on the Vaihingen dataset are presented in Figure 10. For each model, “Detection Errors” represents the visualized maps of error pixels, where blue denotes missed pixels, and green indicates misdetected pixels, while “Predicted” shows the predicted result map of the model. Figure 10(a) illustrates that the proposed BEMS-UNetFormer model significantly improves the accuracy of building boundary segmentation and effectively reduces missed detections. In contrast, UNetFormer and SFA-Net incorrectly classify buildings as white streets in certain regions. In the bottom left corner of Figure 10(b), UNetFormer misclassifies areas of low vegetation as trees, whereas the proposed model and SFA-Net demonstrate better accuracy in distinguishing these closely related categories. Furthermore, Figure 10(c) highlights that the proposed model maintains a low level of missed and misidentified pixels, particularly excelling in identifying small-sized targets like cars. This stands out compared to UNetFormer, which exhibits noticeable missed detections for such targets and often misclassifies them as streets.

###  Ablation experiments

In this section, ablation experiments are conducted on the Potsdam dataset to validate the effectiveness of the proposed modules. Segmentation performance is quantitatively evaluated using metrics such as MF1, OA, and MIoU. Using UNetFormer as the benchmark network, the BAM, BFM, and MSC-ASPP modules are progressively added, and their contributions are analyzed through metric comparisons. Additionally, the impact of different module combinations on the model’s segmentation capability is explored. Since the BFM module relies on boundary information extracted by the BAM module and cannot perform feature fusion independently, its effect was not separately analyzed in these experiments.

Table 4 presents the results of the ablation experiments. Adding the BAM module to the encoding layer improves the MF1, OA, and MIoU metrics by 0.6%, 0.75%, and 1.06%, respectively, demonstrating its ability to better extract shallow contour information. Incorporating the MSC-ASPP module at the encoder-decoder bridge further enhances feature extraction accuracy, with the metrics improving by 0.70%, 0.85%, and 1.20%, respectively. When the BAM module is combined with the BFM module, the metrics increase by 0.57%, 0.76%, and 1.16% compared to the simple feature fusion method. Similarly, introducing the MSC-ASPP module alongside the BAM module enhances performance further, with the metrics improving by 0.74%, 0.86%, and 1.3%. These results indicate that the BAM and MSC-ASPP modules work independently yet mutually reinforce the model’s segmentation capability. Finally, integrating all the proposed modules yields the best segmentation performance, with improvements of 0.8%, 1.02%, and 1.38% in MF1, OA, and MIoU, respectively, compared to the baseline model. These experiments confirm the indispensability of each module in achieving optimal semantic segmentation performance for remote sensing images, particularly in extracting contours and addressing complex feature relationships.

**Table 4:** Ablation experiments on the potsdam dataset.

**Baseline (UNetFormer)**	**BAM**	**BFM**	**MSC-ASPP**	**OA**	**MF1**	**MIoU**
UNetFormer				91.63	90.54	84.74
UNetFormer + BAM	$\surd$			92.23	91.29	85.80
UNetFormer + MSC-ASPP			$\surd$	92.33	91.39	85.94
UNetFormer + BAM + BFM	$\surd$	$\surd$		92.20	91.30	85.90
UNetFormer + BAM + MSC-ASPP	$\surd$		$\surd$	92.37	91.40	86.04
UNetFormer + BAM + BFM + MSC-ASPP	$\surd$	$\surd$	$\surd$	**92.43**	**91.56**	**86.12**

**Fig. 11:**
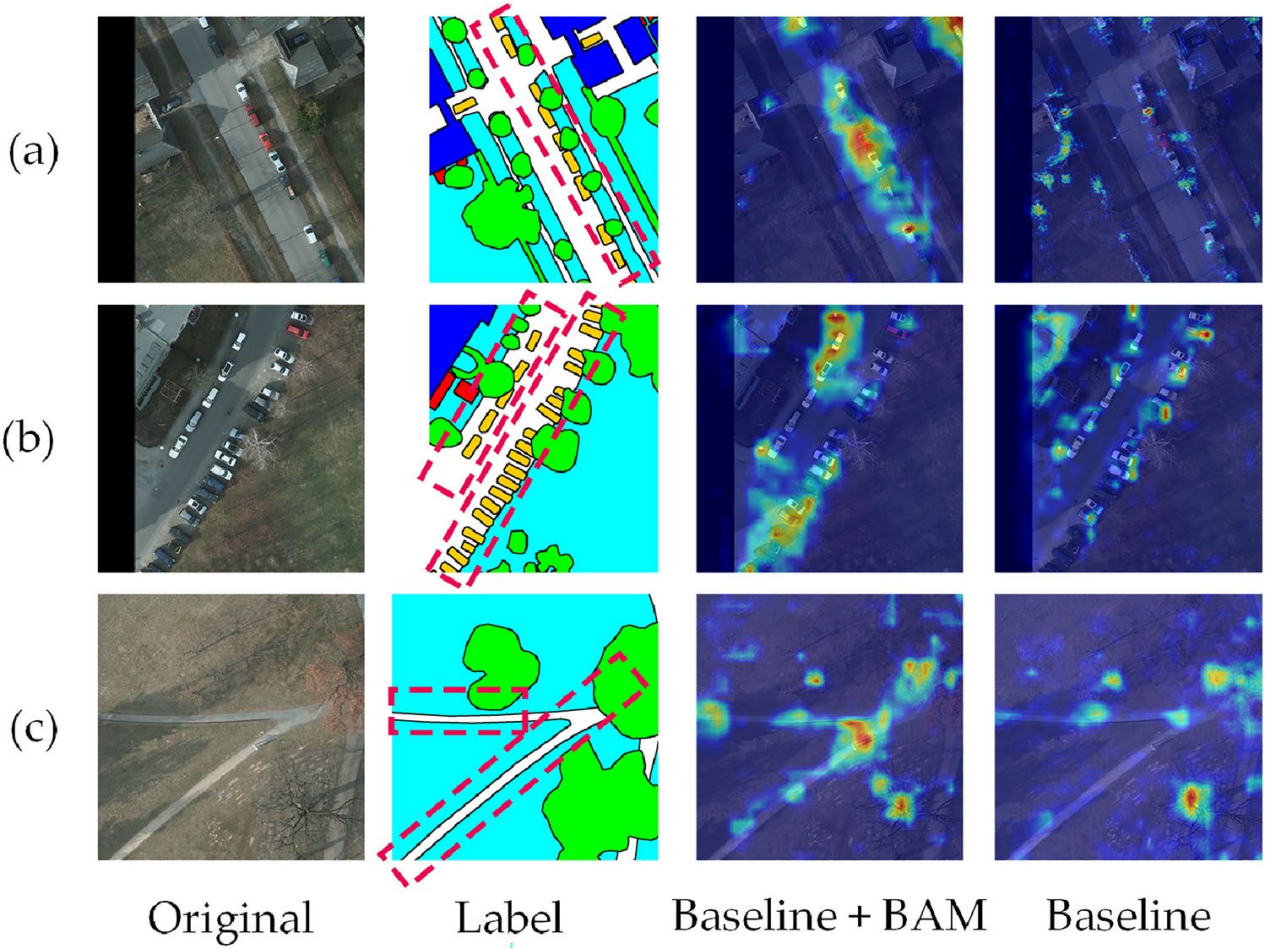
Boundary feature extraction visualization comparison on the potsdam dataset.

To validate the effectiveness of the BAM in target boundary extraction, a comparative analysis was performed on the feature maps generated at the second layer of the network decoder, before and after the module’s integration, using the Potsdam dataset. As illustrated in Figure 11, the figure is divided into four groups: from left to right, they represent the original image, the ground truth, the feature maps produced by the model with the BAM module, and those generated without it. Figure 11(a) demonstrates that integrating the BAM significantly enhances the model’s ability to represent small-scale targets and detailed regions, leading to improved segmentation accuracy. In addition, Figure 11(b) shows that the module greatly improves the model’s capability to capture complex target boundaries, particularly in scenarios involving multiple target categories. Moreover, as shown in Figure 11(c), the feature maps generated by the model with the BAM module exhibit a clear focus on target boundaries, with notably enhanced edge features. In contrast, the feature maps from the model without the module display weaker boundary emphasis, characterized by blurred and less distinct edges. These results confirm the BAM module’s role in improving the precision and robustness of target boundary representation.

###  Experiments of MSC-ASPP

#### Impact of cascaded ASPP on model performance

To analyze the impact of the cascade structure of the ASPP on model performance, this experiment was designed based on the original dilation rate combinations (6, 12, 18, 24) under different cascade operation conditions. The specific results are presented in Table 5.

The experimental results show that the UNetFormer model with ASPP but without cascade operation outperforms the baseline UNetFormer model in terms of IoU and F1 scores for the Street, Building, LowVeg, Tree, and Car categories. The OA increased from 90.54% to 90.82%, the MIoU from 84.74% to 85.09%, and the MF1 from 91.63% to 91.83%. Moreover, the introduction of the cascade operation significantly enhanced the model performance, particularly for the Street and Building categories. The overall accuracy further improved to 91.30%, while the MIoU and MF1 increased to 85.87% and 92.30%, respectively. These results demonstrate that the cascade structure of the ASPP effectively captures multi-scale features, enhancing the model’s ability to parse complex scenes and significantly improving segmentation accuracy, particularly for challenging categories such as Street and Building.

**Table 5:** Experimental results on the impact of the cascade structure of the ASPP module on model performance.

**Parameter**	**Street**		**Building**		**LowVeg**		**Tree**		**Car**		**OA**	**MIoU**	**MF1**
	**IoU**	**F1**	**IoU**	**F1**	**IoU**	**F1**	**IoU**	**F1**	**IoU**	**F1**			
Model 1	63.84	77.94	88.64	93.97	76.83	86.90	78.99	88.26	**91.19**	**95.39**	90.54	84.74	91.63
Model 2	64.93	79.02	89.77	94.50	77.56	87.44	79.54	88.63	90.13	94.36	90.82	85.09	91.83
Model 3	**65.96**	**79.49**	**90.90**	**95.23**	**78.29**	**87.82**	**80.09**	**88.94**	91.07	95.33	**91.30**	**85.87**	**92.30**

Model 1 represents the baseline UNetFormer model; Model 2 represents UNetFormer+ASPP (without cascade structure); Model 3 represents UNetFormer+ASPP (with cascade structure).

#### Experiments with different combinations of dilation rates

To comprehensively explore the multiscale effect, this study investigates the impact of different combinations of dilation rates on the segmentation performance across various target types. Through comparative experiments, the optimal parameter set proposed in this paper is evaluated. Multiple combinations of dilation rates are designed and tested, and the results are analyzed to assess their enhancement effect on segmentation accuracy.

As shown in Table 6, the combination (4, 8, 12, 16) achieves the best performance, with MF1, OA, and MIoU reaching 92.33%, 91.39%, and 85.94%, respectively. Notably, this combination achieves the highest IoU and F1 score for the small target “Car”, with values of 91.49% and 95.55%, demonstrating a significant advantage in detecting small-sized objects. Other combinations, such as (4, 8, 12) and (6, 12, 18, 24), also perform well for most target categories but are less effective in detecting small targets compared to (4, 8, 12, 16). For instance, the combination (6, 12, 18, 24) achieves an MF1 of 91.30%, which is lower than that of (4, 8, 12, 16) across all targets. These findings highlight the effectiveness of the (4, 8, 12, 16) combination in improving segmentation accuracy, particularly for small and complex targets.

**Table 6:** Experimental results for different combinations of dilation rates on the potsdam dataset.

**Parameter**	**Street**		**Building**		**LowVeg**		**Tree**		**Car**		**OA**	**MIoU**	**MF1**
	**IoU**	**F1**	**IoU**	**F1**	**IoU**	**F1**	**IoU**	**F1**	**IoU**	**F1**			
(4, 8, 12)	67.20	80.38	90.17	94.84	77.74	87.47	78.06	87.67	90.54	95.03	90.85	84.99	91.87
(6, 12, 18)	61.49	76.15	90.81	95.18	77.87	87.56	78.35	87.86	91.43	95.52	90.58	85.12	91.85
(6, 12, 18, 24)	65.96	79.49	90.90	95.23	**78.29**	**87.82**	**80.09**	**88.94**	91.07	95.33	91.30	85.87	92.30
(4, 8, 12, 16)	**68.70**	**81.45**	**91.63**	**95.63**	78.03	87.66	79.63	88.66	**91.49**	**95.55**	**91.39**	**85.94**	**92.33**

#### Comparison of MSC-ASPP with similar modules

To validate the effectiveness of the MSC-ASPP, this work compares it with similar modules, including ASPP, Spatial Pyramid Pooling - Fast (SPPF)^48^, and Receptive Field Block (RFB)^49^, using the same training strategies on the Potsdam dataset. The evaluation is conducted based on three metrics: MF1, OA, and MIoU. As shown in Table 7, UNetFormer+MSC-ASPP outperforms the other modules across all three metrics, achieving MF1, OA, and MIoU scores of 92.33%, 91.39%, and 85.94%, respectively, thereby demonstrating the effectiveness of the MSC-ASPP module on the Potsdam dataset.

**Table 7:** Comparison of different modules on the potsdam dataset.

**Model**	**MF1**	**OA**	**MIoU**
UNetFormer + ASPP	91.83	90.82	85.09
UNetFormer + SPPF	91.99	91.13	85.35
UNetFormer + RFB	91.74	90.82	84.93
UNetFormer + MSC-ASPP	**92.33**	**91.39**	**85.94**

**Fig. 12:**
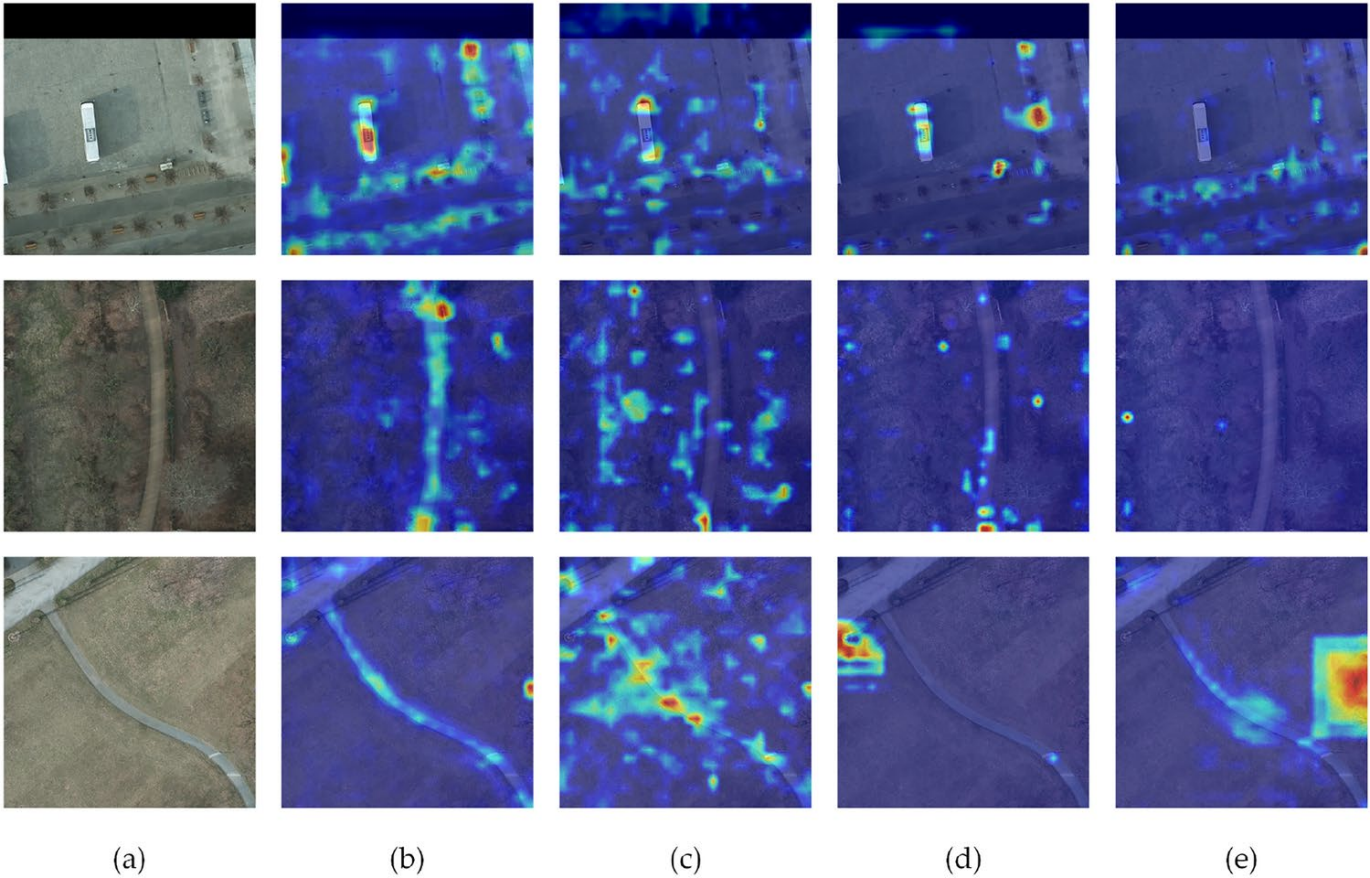
Comparison of heat maps: (**a**) Original, (**b**) MSC-ASPP, (**c**) ASPP, (**d**) SPPF and (**e**) RFB.

To compare the differences in feature extraction among these modules, a visual analysis of feature maps generated by the MSC-ASPP, ASPP, SPPF, and RFB was conducted, as shown in Figure 12. Although the ASPP captures a considerable amount of regional information, its attention is distributed too broadly, failing to effectively focus on the primary target objects, which results in insufficient specificity of feature representation. The SPPF and RFB, while somewhat reducing the dispersion of attention, exhibit overly confined attention areas and fail to fully capture critical target information. This limitation is particularly evident in complex scenarios, where they are prone to missing important object details. In contrast, the proposed MSC-ASPP, through the use of multi-scale cascaded dilated convolutions and a feature fusion mechanism, significantly enhances the discriminative capability of the feature maps. Additionally, by progressively integrating feature information across different scales, the MSC-ASPP further optimizes the spatial distribution of features, making it more effective in capturing key characteristics of objects.

###  Analysis of confusion matrix

**Fig. 13:**
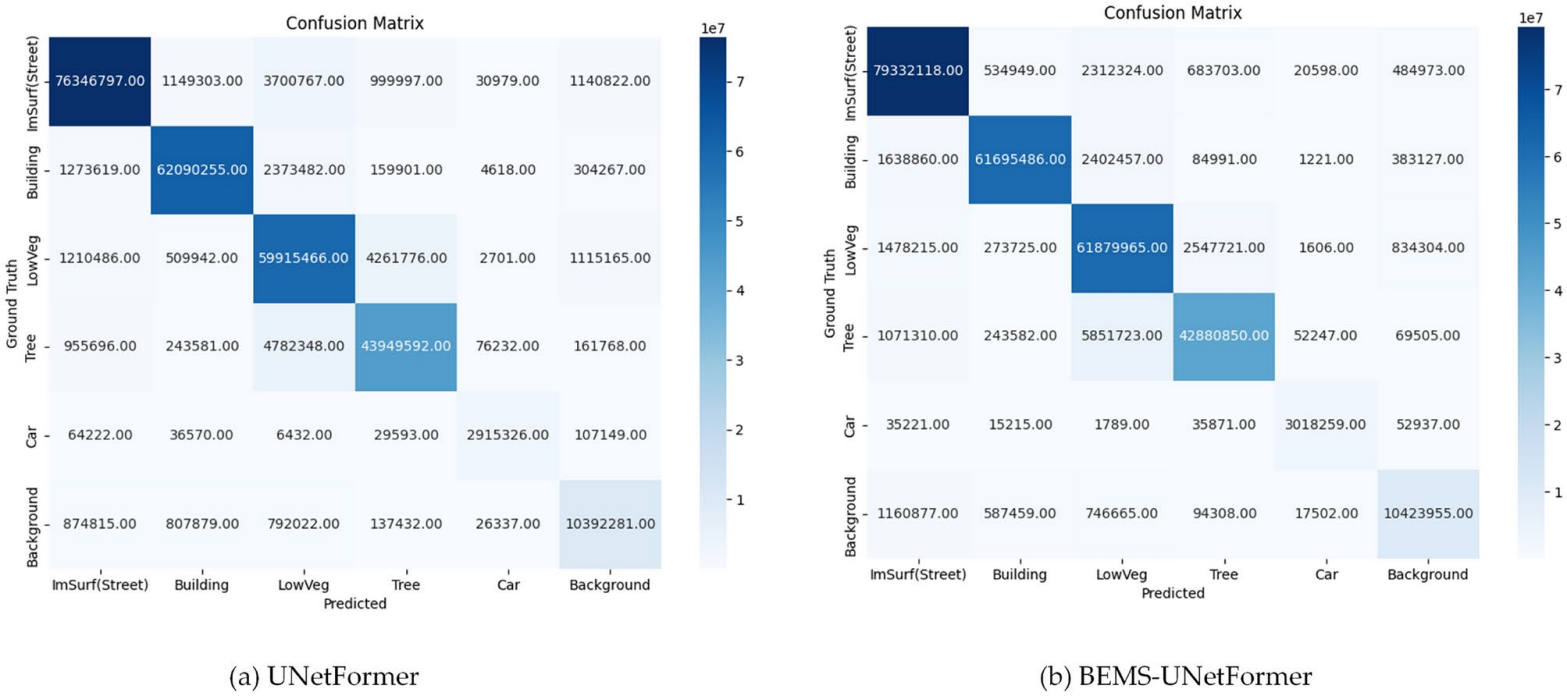
Confusion matrix of UNetFormer and BEMS-UNetFormer segmentation results on the potsdam dataset.

To verify the validity and generalization ability of the proposed model, a comparison was conducted with the base model using confusion matrices on the Potsdam dataset. Figure 13(a) illustrates the segmentation results of BEMS-UnetFormer, while Figure 13(b) presents those of UNetFormer.The analysis of the two confusion matrices reveals an overall improvement in the number of correctly classified instances across the four categories: LowVeg, Background, Car, and imSurf(Street). Notably, the correct detections for the small-sized target “Car” increased from 2,915,326 to 3,018,259, proving the significant enhancement in segmentation accuracy for small-sized targets. Furthermore, the correct detections for “LowVeg” rose from 59,915,466 to 61,879,965, and for street from 76,346,797 to 79,332,118. A detailed analysis of the confusion matrix reveals that, in the baseline model, “Car” were misclassified as “LowVeg” on 6,432 occasions. In the improved BEMS-UnetFormer, this error was reduced to 1,789. Similarly, the number of “Car” misclassified as “Building” decreased from 36,570 to 15,215. These improvements significantly enhanced the segmentation recall rate for the car category. Overall, the improved algorithm substantially optimized the model’s overall performance, demonstrating remarkable effectiveness, particularly in segmenting small-scale targets and easily confusable objects.

###  Experiments of loss function

In this section, the semantic segmentation performance of three different loss functions is compared on the Potsdam dataset: the Improved Loss Function, Boundary Loss, and Hausdorff Distance Loss. The results, shown in Table 8, indicate that the proposed method achieves the best overall performance, with OA, MIoU, and MF1 scores of 91.56%, 86.12%, and 92.43%, respectively.

For specific categories, the proposed method excels in IoU and F1 scores for the Street and Tree categories, highlighting its effectiveness in handling fine boundary segmentation tasks. For the Building and Car categories, it performs comparably to the other loss functions but shows slight improvements. In contrast, the Boundary Loss demonstrates a minor advantage in the LowVeg category; however, its overall performance is inferior to the proposed method.These findings confirm that the Improved Loss Function not only enhances segmentation accuracy but also addresses fine-grained boundary challenges more effectively than the other loss functions tested.

**Table 8:** Experimental results on the impact of different loss functions on model performance.

**Parameter**	**Street**		**Building**		**LowVeg**		**Tree**		**Car**		**OA**	**MIoU**	**MF1**
	**IoU**	**F1**	**IoU**	**F1**	**IoU**	**F1**	**IoU**	**F1**	**IoU**	**F1**			
Boundary Loss	68.78	81.50	91.30	**95.45**	**78.54**	**87.98**	78.95	88.24	91.20	**95.89**	91.46	86.09	92.41
Hausdorff Distance Loss	67.99	80.95	91.21	95.40	78.26	87.80	78.97	88.25	**91.43**	95.52	91.34	85.84	92.27
Ours	**69.36**	**81.91**	**91.58**	94.57	78.46	87.93	**79.34**	**88.48**	91.20	95.57	**91.56**	**86.12**	**92.43**

###  Evaluation of noise robustness in models

The noise robustness of the model is a crucial factor influencing its performance, largely determined by the inherent noise in remote sensing images. This noise arises from two primary sources. The first source is external environmental factors during image capture, such as insufficient lighting, equipment movement, and atmospheric turbulence, which degrade image quality through effects like blurring and streaking. The second source is intrinsic noise generated during image acquisition and processing, such as the mosaic effect in the original image, which significantly interferes with image quality.

Noise impacts model performance in several ways. It can blur features in boundary regions, reducing segmentation accuracy. Additionally, it may cause confusion when recognizing categories with similar visual features (e.g., low vegetation and trees). Lastly, noise obscures the features of small targets, hindering the model’s ability to detect these targets accurately.

**Fig. 14:**
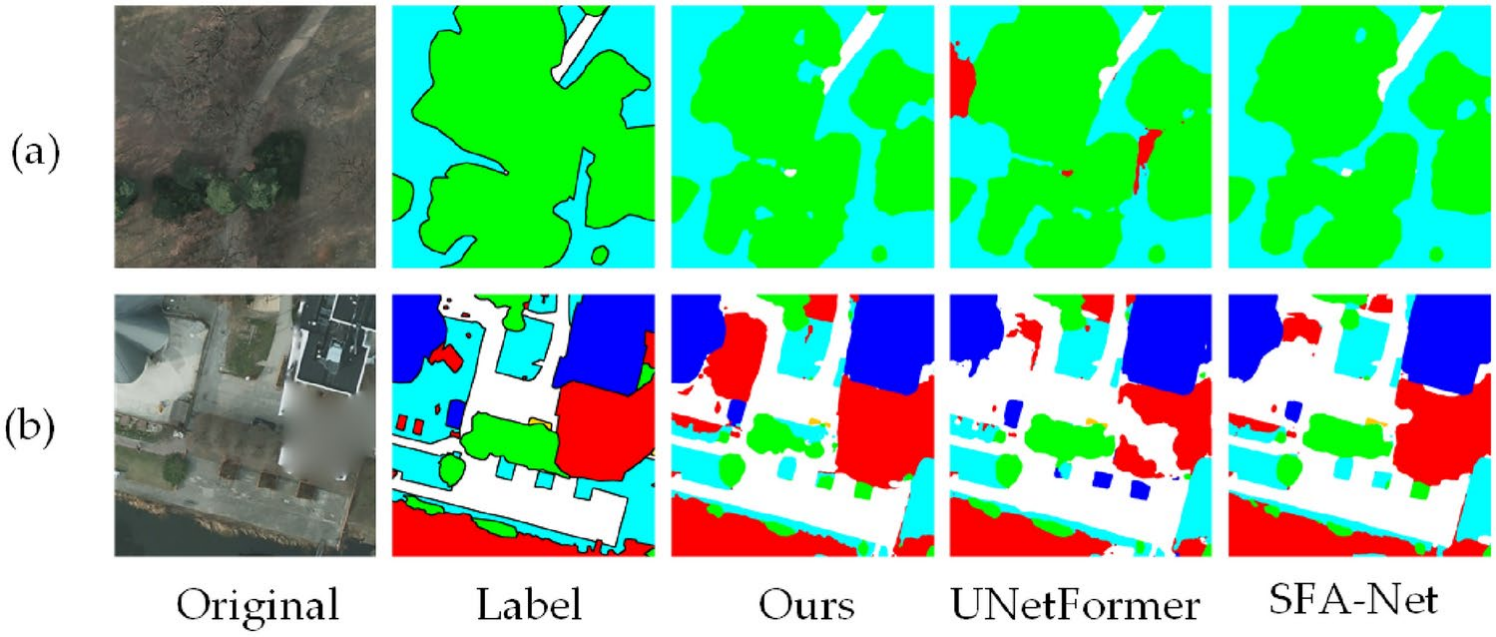
Visualization analysis of model robustness to noise on the potsdam dataset.

In this work, we validate the robustness of the proposed model in noisy environments, with visualization results presented in Figure 14. As shown in Figure 14(a), under conditions of tree silhouette streaks and image blurring, both the proposed model and SFA-Net accurately recognize trees and low vegetation. In contrast, the UNetFormer model misclassifies some trees as background. Similarly, Figure 14(b) illustrates the model’s performance in regions with mosaic noise, specifically in the lower right of the image. While the proposed model correctly identifies the area, even when labeled as background, the UNetFormer model misclassifies it as a white opaque surface. These experimental results confirm the significant advantages of the proposed model in terms of noise robustness. Its ability to handle noise interference in remote sensing images substantially improves recognition accuracy, particularly under challenging conditions.

## Discussion

### Performance evaluation

The performance advantages of BEMS-UNetFormer primarily stem from its design for multi-scale feature extraction and boundary enhancement mechanisms. The BAM uses attention mechanisms to strengthen the extraction of edge features, enabling the model to excel in handling complex boundaries and small-scale objects. For instance, in the Potsdam dataset, BEMS-UNetFormer achieved the highest IoU and F1 scores in the segmentation tasks for vehicles and streets, which can be attributed to the precise edge information captured by the BAM. Furthermore, the MSC-ASPP module employs a combination of atrous convolution rates (4, 8, 12, 16) to effectively aggregate contextual information across multiple scales, thereby enhancing the model’s adaptability to objects of varying sizes. Experimental results indicate that this atrous rate configuration is particularly effective for small-object segmentation, with IoU and F1 scores for vehicles on the Potsdam dataset reaching 91.49% and 95.57%, respectively, significantly outperforming other configurations. Compared to modules such as ASPP, SPPF, and RFB, MSC-ASPP demonstrates the ability to generate more discriminative feature maps, focusing more effectively on target regions and thus improving segmentation accuracy. This design not only preserves rich local structural details but also enhances the representation capability of shallow features, enabling the model to achieve an optimal balance between capturing local details and global contextual information in complex scenarios.

A comparison of parameter counts and computational complexities across models is shown in Table 9. The parameter count affects hardware resource consumption, with fewer parameters requiring less hardware. Similarly, fewer floating-point operations (FLOPS) improve model computational speed. From the perspective of computational efficiency and model optimization, BEMS-UNetFormer demonstrates an advantage with a parameter count of 20.1MB and a computational complexity of 84.2G, outperforming models such as LOGCAN++ in terms of efficiency. However, in practical applications, especially on resource-constrained devices such as mobile terminals or embedded systems, BEMS-UNetFormer may still encounter limitations due to insufficient computational resources. To further enhance the model’s practicality, future work can focus on optimization techniques such as model pruning and knowledge distillation. Model pruning effectively reduces computational complexity and storage demands by removing redundant neurons and parameters. Meanwhile, knowledge distillation transfers the expertise of BEMS-UNetFormer to smaller, more efficient models, significantly improving inference speed while maintaining segmentation accuracy.

**Table 9:** Comparison of parameter counts and computational complexity of models.

**Model**	**FLOPS (G)**	**Para (MB)**
SegFormer	44.30	**3.72**
UNetMamba	100.52	14.76
LOGCAN++	123.69	30.90
SFA-Net	**42.80**	10.70
UNetFormer	47.63	11.40
Ours	84.20	20.10

### Limitations and future works

**Fig. 15:**
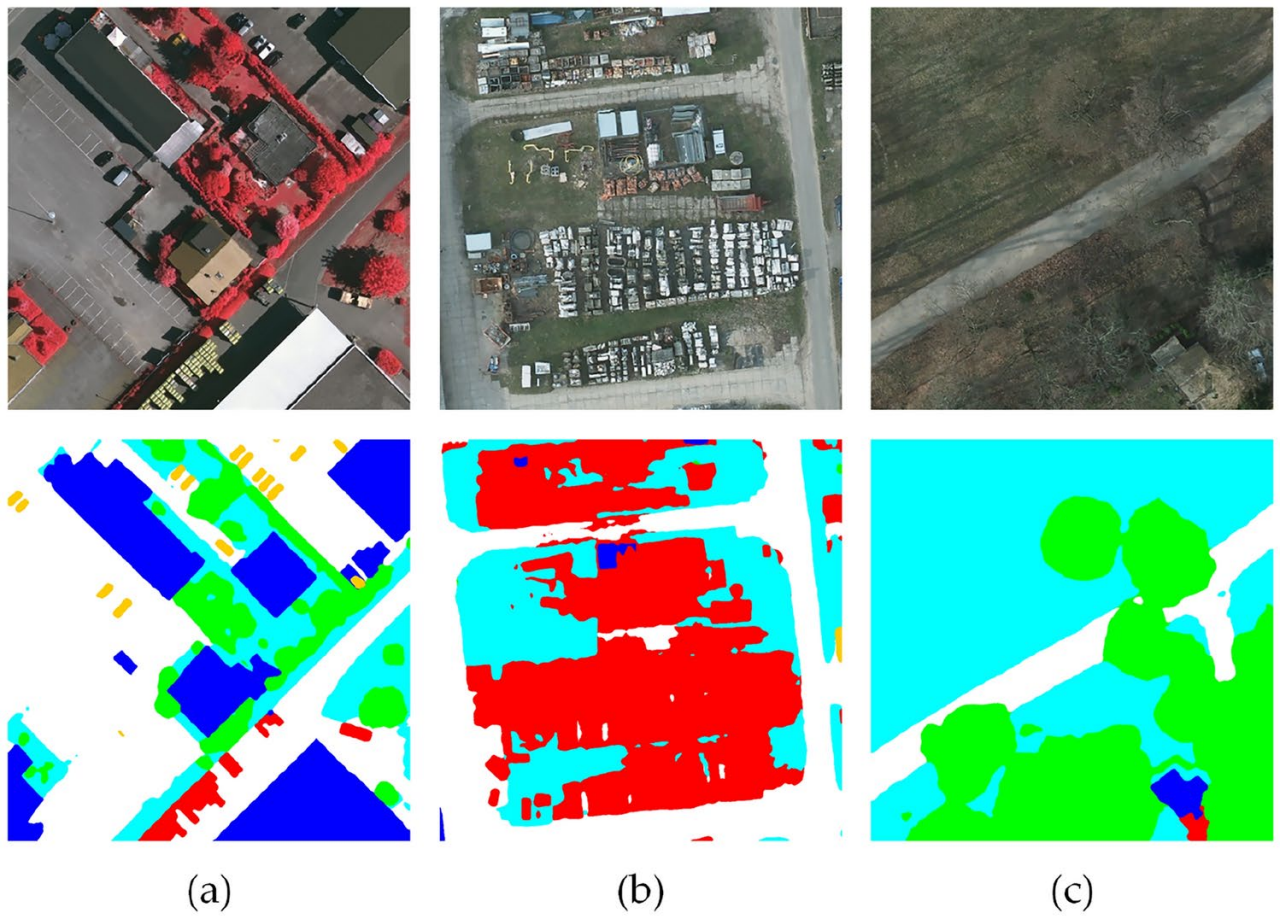
Some misidentified cases: (**a**) Reflection of buildings, (**b**) Large number of consecutive, dense and small objects, (**c**) Residential area obscured by trees.

Although BEMS-UNetFormer demonstrates excellent performance in most scenarios, segmentation errors still occur in certain complex situations, reflecting the model’s technical limitations. For instance, in Figure 15(a), the reflection of trees on the road is misclassified as the background, which might be attributed to insufficient robustness of the model to variations in lighting conditions. Changes in lighting can significantly alter the color and texture of objects in the image, thereby affecting the stability of feature extraction. Additionally, in Figure 15(b), a large number of densely packed small objects are challenging to distinguish, indicating the model’s limitations in handling high-density targets. This could result from the current multi-scale feature fusion strategy failing to sufficiently capture subtle differences between densely packed objects. Future improvements could involve the introduction of more effective multi-scale feature fusion strategies or dynamic attention mechanisms, enabling the model to adaptively focus on objects of varying scales and types, thereby enhancing segmentation accuracy. In Figure 15(c), buildings partially obscured by trees, with colors similar to the trees, are erroneously recognized as the background. This issue could be addressed by incorporating occlusion-aware mechanisms or integrating depth information, such as data from LiDAR, to improve the model’s capability in recognizing occluded targets.

## Conclusions

In this work, a boundary-enhanced semantic segmentation network for multi-scale remote sensing images (BEMS-UNetFormer) is proposed. Built on the original UNetFormer framework, the improved Boundary-Aware Module (BAM) extracts shallow contour information, while the Boundary-Guided Fusion Module (BFM), inspired by attentional mechanisms, enhances the network’s ability to capture edge features. Additionally, Multi-Scale Cascaded Atrous Spatial Pyramid Pooling (MSC-ASPP) is incorporated in the codec bridge to perform multi-scale deep feature extraction, effectively amplifying relevant features and suppressing irrelevant ones. Experimental results on the Potsdam and Vaihingen datasets demonstrate that the proposed method outperforms five classical methods under the same conditions. It achieves higher segmentation accuracy and excels in segmenting small-sized targets and defining target boundaries.

## Data Availability

The datasets analysed during the current study are available at https://www.isprs.org/education/benchmarks/UrbanSemLab/Default.aspx
